# Combined impact of lipidomic and genetic aberrations on clinical outcomes in metastatic castration-resistant prostate cancer

**DOI:** 10.1186/s12916-022-02298-0

**Published:** 2022-03-25

**Authors:** Blossom Mak, Hui-Ming Lin, Edmond M. Kwan, Heidi Fettke, Ben Tran, Ian D. Davis, Kate Mahon, Martin R. Stockler, Karen Briscoe, Gavin Marx, Alison Zhang, Megan Crumbaker, Winston Tan, Kevin Huynh, Thomas G. Meikle, Natalie A. Mellett, Andrew J. Hoy, Pan Du, Jianjun Yu, Shidong Jia, Anthony M. Joshua, David J. Waugh, Lisa M. Butler, Manish Kohli, Peter J. Meikle, Arun A. Azad, Lisa G. Horvath

**Affiliations:** 1 Chris O’Brien Lifehouse, Missenden Rd, Camperdown, New South Wales 2050 Australia; 2 Garvan Institute of Medical Research, Darlinghurst, Sydney, New South Wales Australia; 3 University of Sydney, Sydney, New South Wales Australia; 4 St Vincent’s Clinical School, UNSW, Sydney, New South Wales Australia; 5 Monash Health, Melbourne, Victoria Australia; 6 Peter MacCallum Cancer Centre, Melbourne, Victoria Australia; 7 Sir Peter MacCallum Department of Oncology, University of Melbourne, Melbourne, Victoria Australia; 8 Eastern Health Clinical School, Monash University, Melbourne, Victoria Australia; 9 Eastern Health, Box Hill, Victoria Australia; 10 Royal Prince Alfred Hospital, Camperdown, New South Wales Australia; 11 Concord Repatriation General Hospital, Concord, New South Wales Australia; 12 Mid North Coast Cancer Institute, Coffs Harbour, New South Wales Australia; 13 Sydney Adventist Hospital, Wahroonga, New South Wales Australia; 14 The Kinghorn Cancer Centre, St Vincent’s Hospital, Darlinghurst, New South Wales Australia; 15 Mayo Clinic, Jacksonville, FL USA; 16 Baker Heart and Diabetes Institute, Melbourne, Victoria Australia; 17 School of Medical Sciences, Charles Perkins Centre, Faculty of Medicine and Health, University of Sydney, Camperdown, New South Wales Australia; 18 Predicine, Inc., Hayward, CA USA; 19 Queensland University of Technology, Brisbane, Queensland Australia; 20 Adelaide Medical School and Freemason’s Foundation Centre for Men’s Health, University of Adelaide, Adelaide, South Australia Australia; 21 South Australian Health and Medical Research Institute, Adelaide, South Australia Australia; 22 Huntsman Cancer Institute, Salt Lake City, UT USA; 23 Department of Medicine, School of Clinical Sciences, Monash University, Melbourne, Victoria Australia

**Keywords:** Androgen receptor, Biomarker, Castration-resistant prostate cancer, Genomics, Lipid, PI3K, RB1, TP53

## Abstract

**Background:**

Both changes in circulating lipids represented by a validated poor prognostic 3-lipid signature (3LS) and somatic tumour genetic aberrations are individually associated with worse clinical outcomes in men with metastatic castration-resistant prostate cancer (mCRPC). A key question is how the lipid environment and the cancer genome are interrelated in order to exploit this therapeutically. We assessed the association between the poor prognostic 3-lipid signature (3LS), somatic genetic aberrations and clinical outcomes in mCRPC.

**Methods:**

We performed plasma lipidomic analysis and cell-free DNA (cfDNA) sequencing on 106 men with mCRPC commencing docetaxel, cabazitaxel, abiraterone or enzalutamide (discovery cohort) and 94 men with mCRPC commencing docetaxel (validation cohort). Differences in lipid levels between men ± somatic genetic aberrations were assessed with *t*-tests. Associations between the 3LS and genetic aberrations with overall survival (OS) were examined using Kaplan-Meier methods and Cox proportional hazard models.

**Results:**

The 3LS was associated with shorter OS in the discovery (hazard ratio [HR] 2.15, 95% confidence interval [CI] 1.4-3.3, *p* < 0.001) and validation cohorts (HR 2.32, 95% CI 1.59–3.38, *p* < 0.001). Elevated plasma sphingolipids were associated with *AR*, *TP53, RB1* and PI3K aberrations (*p* < 0.05). Men with both the 3LS and aberrations in *AR, TP53, RB1* or PI3K had shorter OS than men with neither in both cohorts (*p* ≤ 0.001). The presence of 3LS and/or genetic aberration was independently associated with shorter OS for men with *AR, TP53, RB1* and PI3K aberrations (*p* < 0.02). Furthermore, aggressive-variant prostate cancer (AVPC), defined as 2 or more aberrations in *TP53, RB1* and/or *PTEN*, was associated with elevated sphingolipids. The combination of AVPC and 3LS predicted for a median survival of ~12 months. The relatively small sample size of the cohorts limits clinical applicability and warrants future studies.

**Conclusions:**

Elevated circulating sphingolipids were associated with *AR*, *TP53*, *RB1*, PI3K and AVPC aberrations in mCRPC, and the combination of lipid and genetic abnormalities conferred a worse prognosis. These findings suggest that certain genotypes in mCRPC may benefit from metabolic therapies.

**Supplementary Information:**

The online version contains supplementary material available at 10.1186/s12916-022-02298-0.

## Background

Prostate cancer (PC) is the second most frequent cancer and fifth leading cause of cancer death in men worldwide [1]. Therapies such as taxanes, androgen receptor signalling inhibitors (ARSI), poly-ADP ribose polymerase inhibitors (PARPi) and targeted radioisotopes have significantly increased survival in metastatic castration-resistant prostate cancer (mCRPC); however, treatments driven by molecular subtyping are currently limited to PARPi in the setting of DNA repair gene aberrations and pembrolizumab in men with microsatellite instability [2]. Moreover, long-term control of mCRPC requires a combined approach targeting multiple hallmarks of cancer, encompassing the cancer genome, the immune system and metabolic factors including lipid metabolism, all of which contribute to cancer progression and treatment resistance [3].

The genomic landscape of mCRPC is well characterised [4], with numerous somatic genetic aberrations linked to poor clinical outcomes. Androgen receptor (*AR*) aberrations (50–70%), *TP53* aberrations (50%), *PTEN* deletion (30%) and *RB1* deletion (20%) are all associated with shorter time on ARSI [4–7]. *RB1* deletion is also correlated with shorter overall survival (OS) in mCRPC [6]. Aggressive-variant prostate cancer (AVPC), a subset of mCRPC which is rapidly progressive with a poor prognosis, is morphologically heterogenous, but has been shown to correlate with genetic aberrations in two or more of *TP53*, *RB1* and/or *PTEN* [8].

Altered lipid metabolism and its impact on PC is increasingly recognised through epidemiological [9–11] and molecular [12, 13] studies. Lipidomic profiling of plasma from men with PC has demonstrated that the plasma lipid profiles including elevated sphingolipids are associated with a poorer prognosis [14–17]. Ceramide, a key sphingolipid, can be metabolised into sphingosine-1-phosphate (S1P) or sphingomyelin to promote cancer cell growth, tumour metastasis and treatment resistance [18–20]. Elevated circulating sphingolipids, in particular ceramides, are associated with poorer clinical outcomes across the natural history of PC, including metastatic relapse in localised PC, earlier androgen deprivation failure in metastatic hormone-sensitive PC, and shorter OS and radiographic progression-free survival (rPFS) in mCRPC [14–17]. We have derived and validated a poor prognostic 3-lipid signature (3LS) consisting of ceramide(d18:1/24:1), sphingomyelin(d18:2/16:0) and phosphatidylcholine(16:0/16:0) to represent the poor prognostic lipidomic profile [14]. The 3LS is independently associated with shorter OS in men with mCRPC commencing docetaxel in internal and external validation cohorts [14, 15].

A key question arises from lipidomic profiling studies—how do circulating lipid aberrations relate to the genomic landscape of prostate cancer? Genetic alterations can lead to metabolic reprogramming in cancer, and conversely, metabolic dysregulation can be an initiator of malignant cellular transformation [21]. The current study is uniquely placed to address this question and provide insights into new potential therapeutic strategies due to the multidisciplinary approach of parallel metabolic and genomic profiling. Thus, we aimed to assess the relationship between the poor prognostic 3-lipid signature, somatic genetic aberrations and clinical outcomes in mCRPC.

## Methods

### Study population and sample collection

The discovery cohort consisted of 149 men with mCRPC commencing taxanes (docetaxel or cabazitaxel) or ARSI (abiraterone or enzalutamide) at seven sites in New South Wales and Victoria, Australia, who were prospectively enrolled between June 2016 and February 2020. All participants provided written informed consent, with ethics approval obtained from Monash Health Institutional Review Board (15571X) and Royal Prince Alfred Hospital Human Research Ethics Committee (X19-0320). Plasma samples were collected prior to starting treatment according to a standardised blood collection protocol [22].

The validation cohort comprised of 142 men with mCRPC commencing docetaxel at a single US tertiary cancer centre (Mayo Clinic), who were prospectively enrolled between September 2009 to August 2013. Details of ethics approval and sample collection for this cohort have been published previously [23].

### Plasma lipidomic analysis

Lipidomic profiling of plasma samples was performed by liquid chromatography-mass spectrometry as previously described [24]. Data was normalised using the Probabilistic Quotient Normalisation method as outlined previously [15], with final lipid levels transformed to logarithm-2 of pmol/mL for statistical analysis. More detail is provided in Additional File 1: Section S1 [14, 15, 24–26].

### Targeted cfDNA sequencing

Plasma cfDNA extraction, next-generation sequencing and bioinformatics analysis was performed as previously reported [22, 27]. Briefly, extracted cfDNA underwent library preparation, panel-based hybridisation (Predicine, Inc.) and enrichment, followed by paired-end sequencing on the Illumina HiSeq XTen. Somatic point mutations, insertions/deletions and copy number alterations were identified using Predicine’s proprietary GeneRADAR technology and DeepSea machine learning bioinformatics algorithm. Circulating tumour DNA (ctDNA) fraction was calculated as previously described [22]. More detail is included in Additional File 1: Section S2 [7, 22, 23, 27].

### Statistical analysis

Time to rPFS and OS were calculated from the date of treatment commencement to event and censored at date of last follow-up if the event had not occurred. Data regarding rPFS was not available for the validation cohort.

Statistical analyses were performed with R version 4.0.2 and SPSS version 27. Principal components analysis of baseline lipidomic profiles was used to assess whether there were any underlying baseline metabolic differences that could confound our subsequent survival analyses. To determine the presence of the circulating 3LS of poor prognosis, lipidomic datasets were aligned to the original cohort in Lin et al [14] from which the 3LS was derived using the ComBat algorithm (R package sva, v3.34.0) and then calculated as previously described (Additional File 1: Section S1.5) [14, 15]. Somatic gene aberrations were defined as copy number variations or mutations.

Differences in lipid levels between men with or without genetic aberrations were assessed with *t*-tests (R package rstatix, v0.7.0). Survival analyses were performed using Kaplan-Meier methods (R package survival, v3.2-10). Cox regression was used to determine associations between the combined presence of 3LS and genetic aberrations, established clinicopathologic factors (Tables 2 and 3) and OS. Men with mCRPC were grouped according to the biomarker combination of 3LS and genetic aberration in the Cox regression analyses as follows: Group 0 = absence of both 3LS and the genetic aberration, Group 1 = presence of one abnormality (either 3LS or the genetic aberration), and Group 2 = presence of both 3LS and the genetic aberration. *P*-values < 0.05 were considered statistically significant.

## Results

### Study cohorts

In the discovery cohort, 149 men with mCRPC commencing taxanes (27%) or ARSI (73%) underwent plasma lipidomic analysis. Their baseline plasma lipidomic profiles did not show major variations between treatment type (taxane vs ARSI) or line of treatment (first vs second) according to principal components analysis (Additional File 1: Fig S3.1-S3.2).

A subset of the discovery cohort (*n* = 106) had available cfDNA for genomic sequencing (Additional File 1: Fig S4). In the validation cohort, 142 men with mCRPC commencing docetaxel underwent plasma lipidomic analysis. cfDNA for genomic sequencing was available from 94 participants (Additional File 1: Fig S4). The clinical characteristics were consistent between the full cohorts and the subsets with lipidomic and genomic analyses (Additional File 1: Table S5).

### Clinical outcomes by 3-lipid signature

**Fig. 1:**
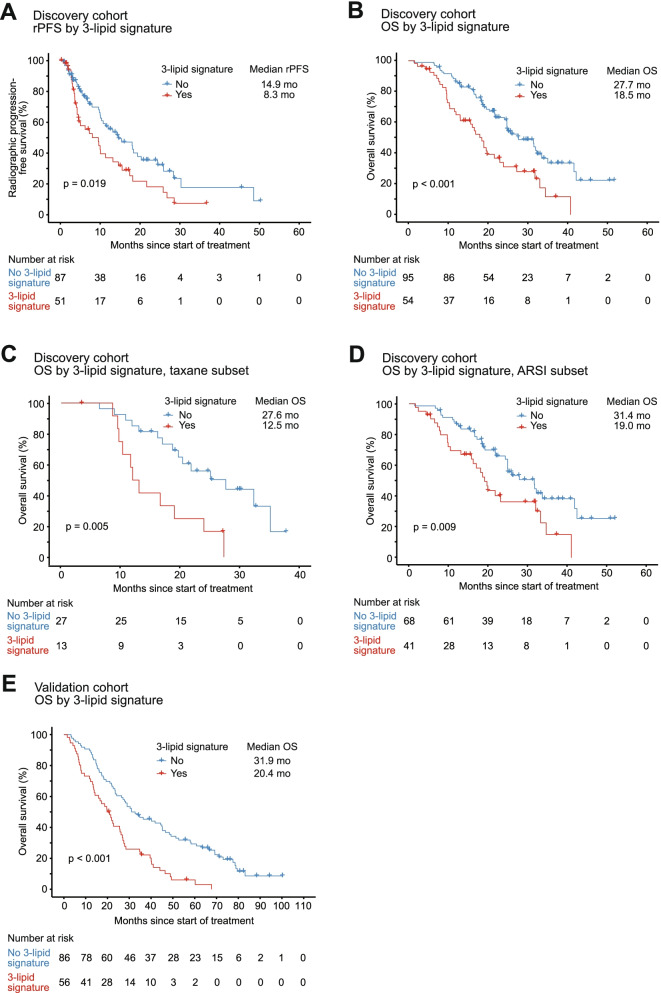
Kaplan-Meier analysis according to 3-lipid signature status in the discovery and validation cohorts. Using **A** radiographic progression-free survival (rPFS) in the discovery cohort, **B** overall survival (OS) in the discovery cohort, **C** OS in the taxane subset of the discovery cohort, **D** OS in the androgen receptor signalling inhibitor (ARSI) subset of the discovery cohort and **E** OS in the validation cohort

The 3LS was present in 41% of men in both cohorts. Consistent with our previous studies [14, 15], the 3LS was associated with shorter rPFS (hazard ratio [HR] 1.71, 95% confidence interval [CI] 1.09–2.66, *p* = 0.019) and OS (HR 2.15, 95% CI 1.4–3.3, *p* < 0.001) in the discovery cohort (Fig. 1A, B). The association with shorter OS remained significant in both the taxane subset (HR 3.29, 95% CI 1.44*–*7.54, *p* = 0.005; Fig. 1C) and ARSI subset (HR 1.99, 95% CI 1.19–3.34, *p* = 0.009; Fig. 1D). Furthermore, the 3LS was associated with shorter OS (HR 2.32, 95% CI 1.59–3.38, *p* < 0.001) in the validation cohort (Fig. 1E).

### Clinical outcomes by genetic aberrations

**Fig. 2:**
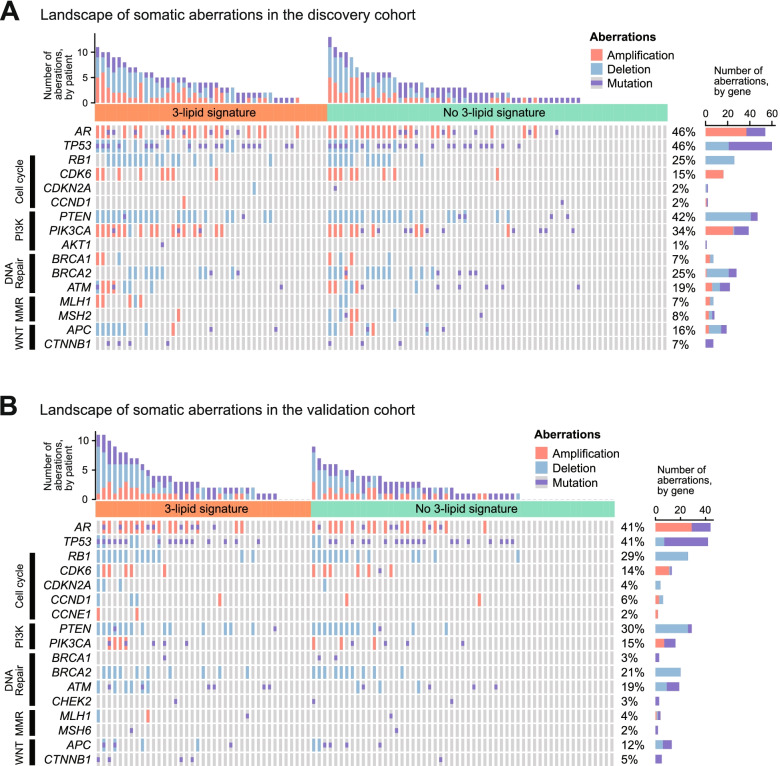
Landscape of somatic aberrations in the **A** discovery cohort and **B** validation cohort

The four most common genetic aberrations seen in both cohorts of men with mCRPC were *AR* aberrations (discovery 46%; validation 41%), *TP53* aberrations (discovery 46%, validation 41%), *RB1* deletion (discovery 25%, validation 29%) and phosphatidylinositol-3-kinase (PI3K) pathway aberrations (discovery 55%, validation 35%) (Fig. 2). All four of these aberrations were associated with shorter OS in the discovery and validation cohorts and with shorter rPFS in the discovery cohort (Table 1).

**Table 1:** Univariable Cox proportional hazards analysis of survival based on genetic aberration in the two cohorts

Genetic aberration	Overall survival in discovery cohort		Overall survival in validation cohort		Radiographic progression-free survival in discovery cohort	
	HR (95% CI)	***P***-value	HR (95% CI)	***P***-value	HR (95% CI)	***P***-value
*AR* aberration	3.87 (2.21–6.77)	**< 0.001**	2.26 (1.43–3.58)	**<0.001**	1.86 (1.07–3.24)	**0.028**
*TP53* aberration	3.55 (2.07–6.08)	**< 0.001**	2.18 (1.37–3.47)	**0.001**	1.80 (1.01–3.13)	**0.039**
*RB1* deletion	4.10 (2.33–7.22)	**< 0.001**	1.79 (1.09–2.94)	**0.021**	3.08 (1.64–5.79)	**< 0.001**
PI3K aberration	2.66 (1.55–4.58)	**< 0.001**	2.11 (1.33–3.34)	**0.002**	2.53 (1.42–4.49)	**0.001**

All *p*-values < 0.05 are highlighted in bold

Data regarding radiographic progression-free survival was not available for the validation cohort

*CI* confidence interval, *HR* hazard ratio, *PI3K* phosphatidylinositol-3-kinase

### The 3-lipid signature and genetic aberrations

The overall frequency of somatic aberrations within the *AR*, *TP53*, cell cycle, PI3K, DNA repair, mismatch repair (MMR) and WNT pathways was increased in men with the 3LS (Fig. 2). In the discovery cohort, 88% of men with the 3LS had ≥ 1 genetic aberration, compared to 75% of men without the 3LS. In total 40% of men with the 3LS had ≥ 5 aberrations, compared to 21% of men without the 3LS. In the validation cohort, 85% of men with the 3LS had ≥ 1 genetic aberration, compared to 69% of men without the 3LS. In total, 26% of men with the 3LS had ≥ 5 aberrations, compared to 15% of men without the 3LS. In both cohorts, increased genomic heterogeneity was associated with the presence of the 3LS.

### Plasma sphingolipids and genetic aberrations

**Fig. 3:**
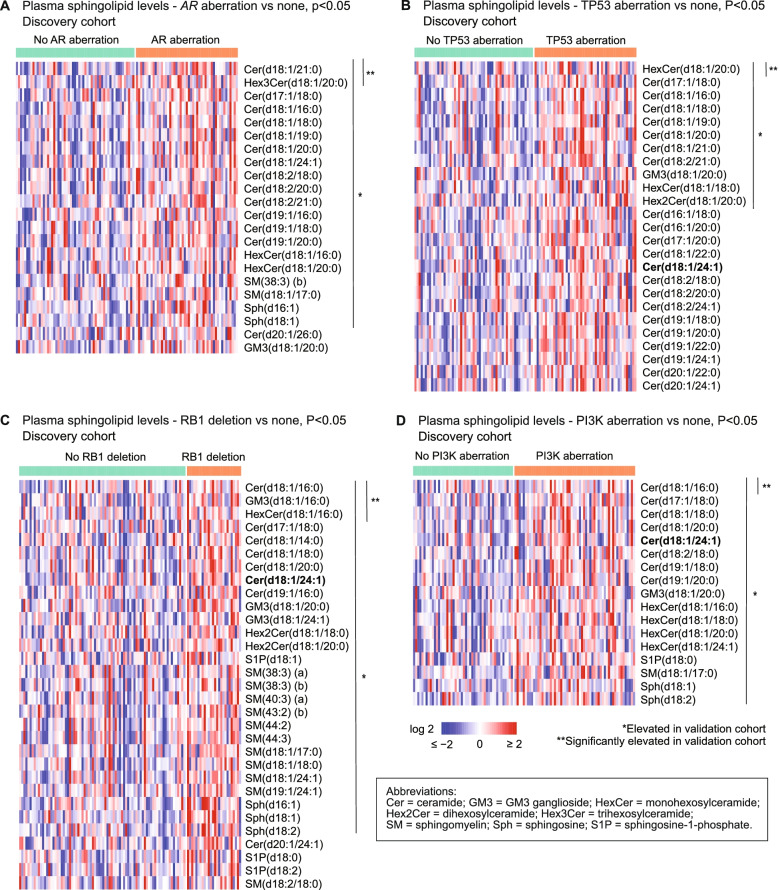
Significantly elevated sphingolipids in men with genetic aberrations in the discovery cohort. Heatmaps show elevated sphingolipids in men with **A** *AR* aberration, **B** *TP53* aberration, **C** *RB1* deletion, and **D** PI3K aberration; compared to men without the aberration. Sphingolipids which are also elevated in the validation cohort are asterisked. Cer(d18:1/24:1), which is a component of the 3-lipid signature, is bolded

Elevated circulating sphingolipids were associated with *AR* aberrations, *TP53* aberrations, *RB1* deletion and PI3K pathway aberrations in both cohorts. In the discovery cohort, 22 sphingolipids were significantly elevated in men with any *AR* aberration compared to men without (*p* < 0.05), including ceramide(d18:1/24:1), a key component of the 3LS (Fig. 3A, Additional File 1: Table S6.1). Twenty of these sphingolipids were also elevated in the validation cohort (Fig. 3A, Additional File 1: Table S6.1). Similarly, 17–31 sphingolipids were significantly elevated in men with *TP53* aberrations, *RB1* deletion or PI3K aberrations (*p* < 0.05), with a proportion of these also elevated in the validation cohort (Fig. 3B–D, Additional File 1: Tables S6.2-6.4).

Aberrations in the DNA repair pathway (*BRCA1/2, ATM, CHEK2*), MMR genes (*MLH1, MSH2, MSH6*) or WNT pathway (*APC, CTNNB1*) were not significantly associated with elevated circulating sphingolipids in either cohort (Additional File 1: Tables S6.5-6.7). This demonstrates that not all genotypes are associated with the poor prognostic metabolic profile.

### 3-lipid signature and genetic aberrations as a biomarker combination

**Fig. 4:**
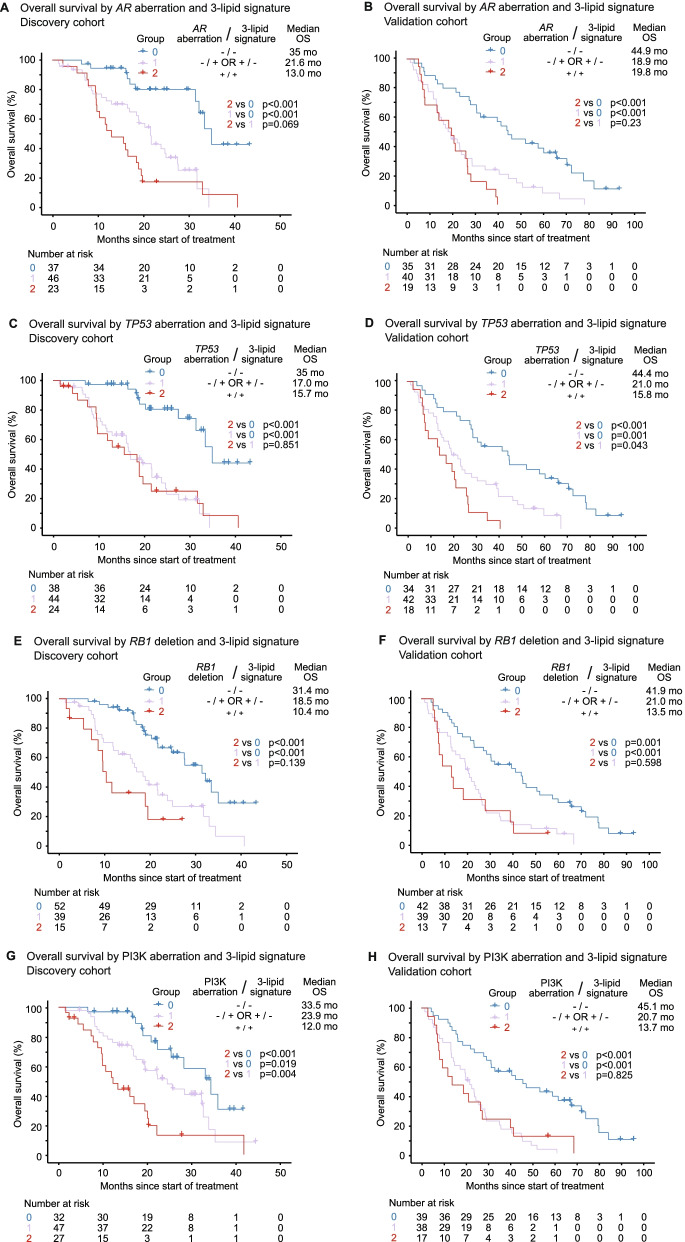
Kaplan-Meier analysis of overall survival (OS) by genetic aberration and 3-lipid signature. OS by *AR* aberration and 3-lipid signature in the **A** discovery and **B** validation cohorts. OS by *TP53* aberration and 3-lipid signature in the **C** discovery and **D** validation cohorts. OS by *RB1* deletion and 3-lipid signature in the **E** discovery and **F** validation cohorts. OS by PI3K aberration and 3-lipid signature in the **G** discovery and **H** validation cohorts

The combined presence of the 3LS with aberrations in *AR*, *TP53*, *RB1* or PI3K in men with mCRPC was associated with poorer prognosis. Men with the 3LS and a genetic aberration (*AR, TP53, RB1* or PI3K) (Group 2) had worse OS than men with neither characteristic (Group 0) in both cohorts (*p* ≤ 0.001; Fig. 4).

In multivariable analysis with clinicopathologic factors, presence of an *AR* aberration and/or the 3LS (Groups 1 and 2) was independently associated with worse OS compared to men with neither characteristic (Group 0) in the discovery cohort (*p* = 0.005; Table 2) and validation cohort (*p* = 0.001; Table 3). The association with shorter OS was also seen with the *TP53* aberration and/or 3LS combination (discovery *p* = 0.001; validation *p* < 0.001)), the *RB1* deletion and/or the 3LS combination (discovery *p* = 0.019; validation *p* < 0.001) and the PI3K and 3LS combination (discovery *p* = 0.009; validation *p* = 0.396) (Tables 2 and 3).

**Table 2:** Cox proportional hazards analysis of overall survival based on biomarker combination in the discovery cohort

	Variable	Univariable Cox regression		Multivariable Cox regression using ***AR*** aberration and 3-lipid signature		Multivariable Cox regression using ***TP53*** aberration and 3-lipid signature		Multivariable Cox regression using ***RB1*** deletion and 3-lipid signature		Multivariable Cox regression using PI3K aberration and 3-lipid signature	
		HR (95% CI)	***P***-value	HR (95% CI)	***P***-value	HR (95% CI)	***P***-value	HR (95% CI)	***P***-value	HR (95% CI)	***P***-value
**Biomarker combinations**	*AR* aberration and/or 3-lipid signature (Groups 1 and 2 vs 0)	4.55 (2.27–9.12)	**< 0.001**	2.89 (1.39–6.00)	**0.005**	–	–	–	–	–	–
	*TP53* aberration and/or 3-lipid signature (Groups 1 and 2 vs 0)	5.22 (2.62–10.41)	**< 0.001**	–	–	3.26 (1.57–6.77)	**0.001**	–	–	–	–
	*RB1* deletion and/or 3-lipid signature (Groups 1 and 2 vs 0)	3.05 (1.79–5.20)	**< 0.001**	–	–	–	–	1.98 (1.12–3.50)	**0.019**	–	–
	PI3K aberration and 3-lipid signature (Group 2 vs 0 and 1)	3.02 (1.75–5.21)	**< 0.001**	–	–	–	–	–	–	2.15 (1.21–3.82)	**0.009**
**Clinicopatholgic factors**	Albumin, g/L^a^	0.86 (0.82–0.91)	**< 0.001**	0.89 (0.84–0.95)	**< 0.001**	0.89 (0.84–0.95)	**< 0.001**	0.89 (0.84–0.95)	**< 0.001**	0.88 (0.83–0.94)	**< 0.001**
	ECOG performance status (≥ 2 vs 0–1)	3.94 (1.75–8.87)	**< 0.001**	1.74 (0.73–4.13)	0.212	1.64 (0.69–3.91)	0.263	1.72 (0.72–4.11)	0.222	2.36 (0.98–5.67)	0.054
	Pain at baseline (present vs absent)	1.89 (1.12–3.19)	**0.018**	1.73 (0.99–3.04)	0.056	1.50 (0.86–2.63)	0.152	1.77 (1.01–3.09)	**0.046**	1.64 (0.93–2.90)	0.089
	Haemoglobin (<90 g/L vs ≥ 90 g/L)	2.70 (0.84–8.73)	0.096	–	–	–	–	–	–	–	–
	PSA, ng/mL^a^	1.00 (1.00–1.00)	0.081	–	–	–	–	–	–	–	–
	ALP, IU/L^a^	1.00 (1.00–1.00)	0.197	–	–	–	–	–	–	–	–
	Treatment type (taxane vs ARSI)	1.05 (0.62–1.79)	0.844	–	–	–	–	–	–	–	–
	Treatment line (second line vs first line)	0.87 (0.51–1.48)	0.597	–	–	–	–	–	–	–	–
	Visceral metastases (present vs absent)	1.59 (0.75–3.37)	0.225	–	–	–	–	–	–	–	–
**ctDNA Fraction**	ctDNA fraction > 2%	1.97 (1.06–3.66)	**0.032**	1.74 (0.91–3.35)	0.097	1.76 (0.93–3.33)	0.083	2.22 (1.17–4.18)	**0.014**	2.24 (1.18–4.24)	**0.014**

Univariable and multivariable Cox regression based on biomarker combination, clinicopathologic factors and ctDNA fraction. Only variables with *p* < 0.05 in univariable analysis were included in multivariable analysis

Group 0 = absence of both 3LS and the genetic aberration; Group 1 = presence of one abnormality (either 3LS or the genetic aberration); Group 2 = presence of both 3LS and the genetic aberration

*ALP* alkaline phosphatase, *ARSI* androgen receptor signalling inhibitor, *CI* confidence interval, *ctDNA* circulating tumour DNA, *ECOG* Eastern Cooperative Oncology Group, *HR* hazard ratio, *PI3K* phosphatidylinositol-3-kinase, *PSA* prostate-specific antigen

All *p*-values < 0.05 are highlighted in bold

^a^Continuous variable

**Table 3:** Cox proportional hazards analysis of overall survival based on biomarker combination in the validation cohort

	Variable	Univariable Cox regression		Multivariable Cox regression using ***AR*** aberration and 3-lipid signature		Multivariable Cox regression using ***TP53*** aberration and 3-lipid signature		Multivariable Cox regression using ***RB1*** deletion and 3-lipid signature		Multivariable Cox regression using PI3K aberration and 3-lipid signature	
		HR (95% CI)	***P***-value	HR (95% CI)	***P***-value	HR (95% CI)	***P***-value	HR (95% CI)	***P***-value	HR (95% CI)	***P***-value
**Biomarker combinations**	*AR* aberration and/or 3-lipid signature (Groups 1 and 2 vs 0)	2.92 (1.79–4.75)	**< 0.001**	2.45 (1.42–4.20)	**0.001**	–	–	–	–	–	–
	*TP53* aberration and/or 3-lipid signature (Groups 1 and 2 vs 0)	2.77 (1.67–4.61)	**< 0.001**	–	–	2.82 (1.64–4.83)	**< 0.001**	–	–	–	–
	*RB1* deletion and/or 3-lipid signature (Groups 1 and 2 vs 0)	2.53 (1.58–4.07)	**< 0.001**	–	–	–	–	1.95 (0.98–3.86)	**< 0.001**	–	–
	PI3K aberration and 3-lipid signature (Group 2 vs 0 and 1)	1.78 (1.03–3.10)	**0.04**	–	–	–	–	–	–	1.32 (0.70–2.50)	0.396
**Clinicopatholgic factors**	PSA, ng/mL^a^	1.00 (1.00–1.00)	**< 0.001**	1.00 (1.00–1.00)	**< 0.001**	1.00 (1.00–1.00)	**< 0.001**	1.00 (1.00–1.00)	**< 0.001**	1.00 (1.00–1.00)	**< 0.001**
	ALP, IU/L^a^	1.00 (1.00–1.00)	**0.002**	1.00 (1.00–1.00)	**0.024**	1.00 (1.00–1.00)	**0.03**	1.00 (1.00–1.00)	0.069	1.00 (1.00–1.00)	**0.034**
**ctDNA Fraction**	ctDNA fraction > 2%	1.91 (1.18–3.08)	**0.008**	1.29 (0.76–2.17)	0.347	1.37 (0.81–2.30)	0.238	1.74 (1.05–2.87)	0.062	1.67 (1.01–2.78)	**0.048**

Univariable and multivariable Cox regression based on biomarker combination, clinicopathologic factors and ctDNA fraction. Only variables with *p* < 0.05 in univariable analysis were included in multivariable analysis

Group 0 = absence of both 3LS and the genetic aberration; Group 1 = presence of one abnormality (either 3LS or the genetic aberration); Group 2 = presence of both 3LS and the genetic aberration

*ALP* alkaline phosphatase, *CI* confidence interval, *ctDNA* circulating tumour DNA, *HR* hazard ratio, *PI3K* phosphatidylinositol-3-kinase, *PSA* prostate-specific antigen

All *p*-values < 0.05 are highlighted in bold

^a^Continuous variable

### 3-lipid signature and aggressive-variant prostate cancer

**Fig. 5:**
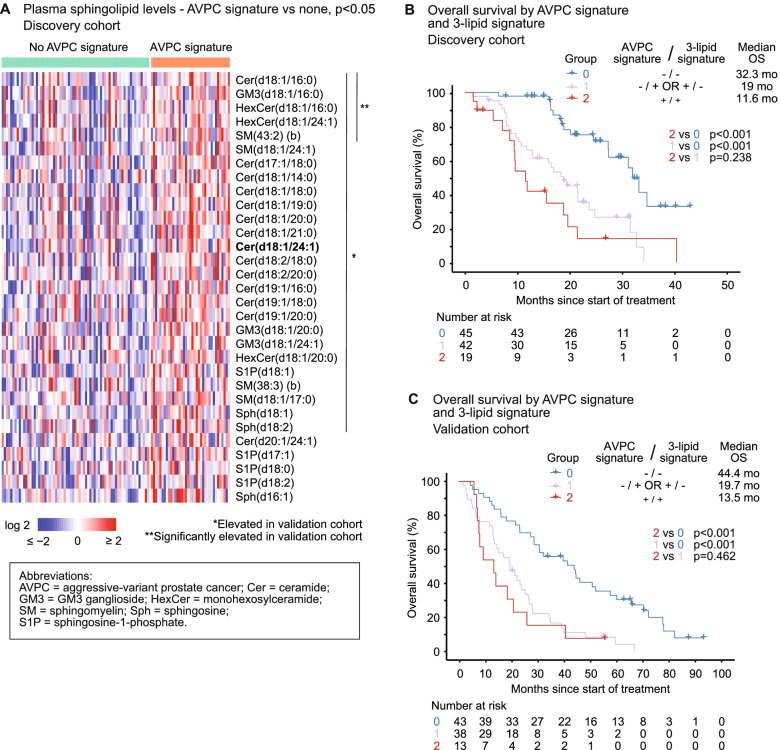
Association between elevated sphingolipids and aggressive-variant prostate cancer (AVPC), and their combined impact on clinical outcomes. **A** Heatmap of sphingolipids with significantly elevated levels in men with the AVPC signature, compared to men without. Sphingolipids which are also elevated in the validation cohort are asterisked. Cer(d18:1/24:1), which is a component of the 3-lipid signature, is bolded. Kaplan-Meier analysis of overall survival by molecular AVPC signature and 3-lipid signature in **B** the discovery cohort and **C** the validation cohort

AVPC, as defined by the presence of genetic aberrations in two or more of *TP53, RB1* and/ or *PTEN* [8], was present in 35% of men in the discovery cohort and 27% of men in the validation cohort. As expected, men with the molecular AVPC signature had shorter OS (discovery: HR 3.57, 95% CI 2.12–6.00, *p* < 0.001; validation: HR 2.40, 95% CI 1.44–3.98, *p* < 0.001). Elevated circulating sphingolipids were associated with AVPC in both cohorts (Fig. 5A).

Men with the combination of 3LS and AVPC had significantly shorter OS in both cohorts, with median survival of ~12 months compared to > 2 years for men with neither signature (discovery 11.6 months vs 32.3 months, *p* < 0.001; validation 13.5 months vs 44.4 months, *p* < 0.001) (Fig. 5B, C). The genetic aberrations of AVPC are the main drivers of poor prognosis in these men, as men with AVPC (regardless of the presence of 3LS) have shorter OS than non-AVPC men with 3LS (Additional File 1: Fig S7.5). However, in both cohorts, a higher proportion of men with AVPC have the 3LS compared to men without AVPC (52% versus 35–39%). The molecular AVPC signature and/or 3LS was independently associated with shorter OS in multivariable analysis with clinicopathological features (Additional File 1: Tables S8.1-S8.2).

## Discussion

This study provides new insights into the correlation between circulating lipids and tumour genotypes in mCRPC. Elevated circulating sphingolipids were correlated with *AR*, *RB1* and PI3K aberrations in two independent cohorts and associated with *TP53* aberrations in the discovery cohort, but not with DNA repair, MMR or WNT pathway aberrations. The presence of a somatic aberration (*AR, TP53, RB1*, PI3K) and/or the 3LS was independently associated with shorter OS in multivariable analysis in both cohorts. In addition, AVPC was associated with elevated sphingolipids and the combination of AVPC and 3LS predicted for a shorter median survival of ~12 months. Taken together, these findings provide the first understanding into the correlation between circulating lipid and somatic gene alterations in mCRPC and suggest that men with *AR, TP53, RB1*, PI3K and AVPC aberrations are more likely to benefit from therapies targeting sphingolipid metabolism than men with DNA repair, MMR or WNT pathway anomalies.

A key question arising from this data is how the genomic and lipidomic changes are related biologically. Not all genotypes are associated with the sphingolipid profiles, suggesting that certain genetic aberrations (*AR, TP53, RB1*, PI3K) are more likely to be dysregulating lipid metabolism. Bivariate analysis of the genotypes with the 3LS was inconclusive regarding whether the genetics was driving the lipid metabolism or vice versa (Additional File 1: Tables S9.1-9.4).

There is emerging evidence of a biological link between lipid metabolism and genetic aberrations including *AR*, *RB1* and PI3K on PC growth. Enhanced ceramide-S1P signalling may be mediating ARSI resistance induced by *AR* gain, as men with mCRPC had significantly shorter ARSI treatment duration if their tumours had *AR* gain in combination with increased expression of sphingolipid genes (involved in ceramide-S1P signalling) [16]. Furthermore, de novo resistance to enzalutamide in androgen-independent cells can be reversed with sphingosine kinase inhibitors in vitro [16]. A recent study demonstrated that a novel fatty acid synthase inhibitor antagonised CRPC growth through metabolic reprogramming and inhibited expression of *AR* and its variants, including AR-V7 [28]. Another study found that 3-hydroxy-3-methyl-glutaryl–CoA reductase (HMGCR), a key enzyme in the cholesterol synthesis pathway, was elevated in enzalutamide-resistant PC cell lines and that simvastatin, a HMGCR inhibitor, blocked *AR* synthesis and inhibited growth in vitro and in vivo [29]. There is also recent evidence suggesting a role for *RB1* in regulating sphingolipid metabolism in advanced PC. In an in vitro model of mCRPC with *RB1* deletion utilising the androgen-independent PC cell line C4-2 after RB knockdown, 27% of upregulated genes were involved in metabolic pathways, including sphingolipid metabolism [30]. In addition, ceramide and its metabolite S1P play key roles in the PI3K pathway, as a negative regulator and activator respectively [31]. Overall, these studies suggest that lipid metabolism may play a biological and possibly clinically relevant role in some of these molecular pathways.

These findings raise the question of whether manipulation of the lipidome might be an effective therapeutic strategy in addition to existing drugs, particularly for patients with unfavourable lipidomic and somatic genetic aberrations. For example in the IPATential150 study, men with mCRPC with PTEN-loss tumours had improved rPFS on treatment with abiraterone and an AKT inhibitor versus abiraterone alone (18.5 months vs 16.5 months, *p* = 0.034) [32]. Our data supports the hypothesis that the addition of a metabolic modifier in men with both a PI3K aberration and 3LS may increase the efficacy of the AKT inhibitor, and this warrants further investigation.

Metabolic therapies are currently not the standard of care for PC, but are used in the treatment of other diseases such as cardiovascular disease and diabetes. Non-pharmacological interventions such as exercise and diet can reduce ceramides [33, 34]. The cholesterol lowering drugs statins and PCSK9 inhibitors reduce levels of circulating ceramides and other sphingolipids in patients with dyslipidaemia [35, 36]. Several studies have already indicated that statin therapy is associated with improved OS in PC [37–40]; however, blood cholesterol levels were not associated with prognosis in prostate cancer [41–43]. Therefore, the beneficial effects of statin therapy may be related to other lipids such as ceramides given that statin can lower the circulating levels of these lipids.

Drugs specifically targeting sphingolipid metabolism are mostly in pre-clinical development in cancer [18], but a specific inhibitor of sphingosine kinase 2, Opaganib (ABC294640), has been tested in patients with solid tumours [44]. Recently, we showed that Opaganib can overcome de novo enzalutamide resistance in androgen-independent PC cells in vitro [16]. Opaganib is currently undergoing a Phase 2 study in combination with AR inhibitors in patients with mCRPC (NCT04207255). Clinical trials are required to determine if repurposing statins or other sphingolipid-targeting therapies for prostate cancer in combination with standard of care can improve clinical outcomes.

Furthermore, plasma lipidomic signatures predicting for high-risk patients in cardiovascular disease already exist [45] and have been translated into a rapid-turnover plasma ceramide test by the Mayo Clinic Laboratories [46]. Overall, the availability of metabolic therapies that can target sphingolipid metabolism in combination with genotyping and lipidomic analysis for patient selection, demonstrates the feasibility of personalised metabolic therapy in a clinical setting for men with mCRPC.

A strength of this study is the analysis of two independent cohorts of prospectively enrolled men with mCRPC using the previously validated 3LS [14, 15], with similar observations in both the discovery and validation cohorts. This study was limited by exclusion of men without available cfDNA for sequencing (29% of discovery cohort, 34% of validation cohort). High levels of ctDNA are associated with poorer clinical outcomes and likely to represent a higher tumour burden [7]. Therefore, the exclusion of men with undetectable/low cfDNA from our cohorts may have resulted in the cohorts having worse clinical outcomes compared to the average population of men with mCRPC. Inadvertent skewing of the cohort characteristics might account for differences between the discovery and validation cohorts (e.g. less significant differences in sphingolipid levels between men with and without genetic aberrations in the validation cohort). The cohorts also had differences in baseline PSA levels and Gleason grade—the median PSA levels were two times higher in the discovery cohort (30 vs 14 ng/mL), and a higher proportion of the discovery cohort had Gleason Grade ≥9 (49% vs 32%). Overall, the relatively small sample size of the cohorts limits clinical applicability and will need to be addressed in the future with studies of larger cohorts. Another potential confounder was the presence of co-occurring genetic aberrations.

## Conclusion

Elevated circulating sphingolipids, especially ceramides, were associated with *AR*, *TP53*, *RB1*, PI3K and AVPC aberrations in mCRPC, and the combination of lipid and genetic abnormalities conferred a worse prognosis. This suggests that approaches targeting the aberrant lipid metabolism defined by the 3LS should be considered in men with these mCRPC genotypes in prospective clinical trials.

## Supplementary Information

**Additional file 1.** Supplementary information for ‘Combined impact of lipidomic and genetic aberrations on clinical outcomes in metastatic castration-resistant prostate cancer’. Supplementary information, figures and tables for ‘Combined impact of lipidomic and genetic aberrations on clinical outcomes in metastatic castration-resistant prostate cancer’.

## Data Availability

The datasets used and/or analysed during the current study are available from the corresponding author on reasonable request.

## Abbreviations

AR: Androgen receptor
ARSI: Androgen receptor signalling inhibitors
AVPC: Aggressive-variant prostate cancer
cfDNA: Cell-free DNA
ctDNA: Circulating tumour DNA
CI: Confidence interval
HMGCR: 3-Hydroxy-3-methyl-glutaryl–CoA reductase
HR: Hazard ratio
mCRPC: Metastatic castration-resistant prostate cancer
MMR: Mismatch repair
OS: Overall survival
PARPi: Poly-ADP ribose polymerase inhibitors
PC: Prostate cancer
PI3K: Phosphatidylinositol-3-kinase
rPFS: Radiographic progression-free survival
S1P: Sphingosine-1-phosphate
3LS: 3-lipid signature
