# Supplementary material for: Combined impact of lipidomic and genetic aberrations on clinical outcomes in metastatic castration-resistant prostate cancer
Source: BMC Med. 2022 Mar 25;20:112. doi: 10.1186/s12916-022-02298-0 (PMC8953070; doi:10.1186/s12916-022-02298-0)
Supplement: Supplementary file 1 — Additional file 1. Supplementary information for ‘Combined impact of lipidomic and genetic aberrations on clinical outcomes in metastatic castration-resistant prostate cancer’. Supplementary information, figures and tables for ‘Combined impact of lipidomic and genetic aberrations on clinical outcomes in metastatic castration-resistant prostate cancer’. [file 12916_2022_2298_MOESM1_ESM.pdf]

**Supplementary Information**

## Table of Contents

S1 Detailed methods for lipidomic analysis ..... 3

S1.1 Blood collection and lipid extraction ..... 3

S1.2 Quality control samples ..... 3

S1.3 Liquid chromatography-mass spectrometry analysis ..... 3

S1.4 Data normalisation ..... 3

S1.5 Dataset alignment and calculation of the 3-lipid signature ..... 4

S2 Detailed methods for targeted cfDNA sequencing ..... 5

S2.1 Blood collection and cfDNA extraction ..... 5

S2.2 Targeted capture, sequencing and bioinformatics ..... 5

Library preparation, hybrid-capture and sequencing ..... 5

Somatic mutation identification ..... 6

Estimation of ctDNA fraction ..... 6

Targeted copy number analysis ..... 6

S2.3 Analytical validation of cfDNA assay CNV detection ..... 6

S3 Principal components analysis (PCA) of baseline plasma lipidomic profiles in the discovery cohort 7

Fig S3.1: PCA of baseline plasma lipidomic profiles in the discovery cohort, samples labelled by treatment type ..... 7

Fig S3.2: PCA of baseline plasma lipidomic profiles in the discovery cohort, samples labelled by treatment line ..... 7

Fig S4: CONSORT diagram ..... 8

Table S5: Patient characteristics of the discovery and validation cohorts ..... 9

S6 Fold difference in lipid levels between men with and without genetic aberrations, as assessed with t-tests ..... 10

Table S6.1: Sphingolipids with significantly elevated levels in men with any *AR* aberration in the discovery cohort compared to men without, and their fold-change in the validation cohort ..... 10

Table S6.2: Sphingolipids with significantly elevated levels in men with any *TP53* aberration in the discovery cohort compared to men without, and their fold-change in the validation cohort ..... 11

Table S6.3: Sphingolipids with significantly elevated levels in men with *RB1* deletion in the discovery cohort compared to men without, and their fold-change in the validation cohort ..... 12

Table S6.4: Sphingolipids with significantly elevated levels in men with any PI3K pathway aberration in the discovery cohort compared to men without, and their fold-change in the validation cohort. ..... 13

Table S6.5: Sphingolipids with significantly elevated levels in men with any DNA repair aberration (*BRCA1/2, ATM, CHEK2*) in the discovery cohort compared to men without, and their fold-change in the validation cohort ..... 13

Table S6.6: Sphingolipids with significantly elevated levels in men with any mismatch repair (MMR) aberration (MLH1, MSH2, MSH6) in the discovery cohort compared to men without, and their fold-change in the validation cohort. .......... 14

Table S6.7: Sphingolipids with significantly elevated levels in men with any WNT pathway aberration (*APC, CTNNB1*) in the discovery cohort compared to men without, and their fold-change in the validation cohort. .......... 14

S7 Kaplan Meier analysis of overall survival by aberration and 3-lipid signature in the discovery and validation cohorts. .......... 15

Fig S7.1: Kaplan Meier analysis of overall survival by *AR* aberration and 3-lipid signature in (A) the discovery cohort and (B) the validation cohort. .......... 15

Fig S7.2: Kaplan Meier analysis of overall survival by *TP53* aberration and 3-lipid signature in (A) the discovery cohort and (B) the validation cohort. .......... 15

Fig S7.3: Kaplan Meier analysis of overall survival by *RB1* deletion and 3-lipid signature in (A) the discovery cohort and (B) the validation cohort. .......... 16

Fig S7.4: Kaplan Meier analysis of overall survival by *PI3K* aberration and 3-lipid signature in (A) the discovery cohort and (B) the validation cohort. .......... 16

Fig S7.5: Kaplan Meier analysis of overall survival by AVPC signature and 3-lipid signature in (A) the discovery cohort and (B) the validation cohort. .......... 17

S8 Cox proportional hazards analysis of overall survival based on the aggressive-variant prostate cancer and 3-lipid signature combination .......... 18

Table S8.1: Cox proportional hazards analysis of overall survival based on the aggressive-variant prostate cancer and 3-lipid signature combination, clinicopathologic factors and ctDNA fraction in the discovery cohort .......... 18

Table S8.2: Cox proportional hazards analysis of overall survival based on the aggressive-variant prostate cancer and 3-lipid signature combination, clinicopathologic factors and ctDNA fraction in the validation cohort .......... 19

S9 Bivariable Cox proportional hazard analyses .......... 20

Table S9.1: Bivariable Cox proportional hazards analysis of overall survival based on 3-lipid signature and *AR* aberration .......... 20

Table S9.2: Bivariable Cox proportional hazards analysis of overall survival based on 3-lipid signature and *TP53* aberration .......... 20

Table S9.3: Bivariable Cox proportional hazards analysis of overall survival based on 3-lipid signature and *RB1* deletion .......... 20

Table S9.4: Bivariable Cox proportional hazards analysis of overall survival based on 3-lipid signature and PI3K aberration .......... 21

S10 Plasma concentrations of sphingolipids in the discovery and validation cohorts. .......... 22

Fig S10.1: Abundance of ceramides in the plasma (pre-treatment) of the Discovery and Validation cohorts. .......... 22

Table S10.1: Concentration range of sphingolipids in plasma (baseline) of the Discovery and Validation cohorts. .......... 23

# S1 Detailed methods for lipidomic analysis

## S1.1 Blood collection and lipid extraction

Peripheral blood from patients was sampled and lipid extraction performed as previously described. Whole blood was collected into 10mL EDTA-containing tubes and two-step centrifugation performed to separate plasma and buffy coat. The centrifugation speed differed between the two cohorts. For the discovery cohort, blood samples were first centrifuged at 1600 x g for 15 minutes and the supernatant transferred to a fresh tube, where it was centrifuged again at 5000 x g for 10 min. For the validation cohort, both centrifugation speeds were 3000 rpm for 10 minutes. Aliquots of the plasma after the second centrifugation were stored at -80$^{o}$C until required.

10µL of plasma was used to extract lipids using a butanol/methanol extraction method. Internal standards were added to the plasma prior to lipid extraction, in order to calculate the concentration of the lipids [lipid concentration = (area of analyte/area of corresponding internal standard) x concentration of internal standard x response factor]. Internal standards and response factors are listed in Huynh *et al* (2019).

## S1.2 Quality control samples

Replicates of two types of quality controls (QC) were extracted and ran together with the study plasma samples:

- Pooled human plasma from healthy individuals (PQC)
- National Institute of Standards and Technology human plasma standard reference material 1950 (NIST1950). This was developed by NIST from a collaboration with the National Institute of Health (NIH), and the National Institute of Diabetes and Digestive and Kidney Disease (NIDDK), to allow comparisons between data sets run within the same laboratory and with other laboratories globally.

The coefficient of variation (%CV) of the lipid levels in these QC samples passed the required threshold of mean %CV <15% and median %CV <10%.

## S1.3 Liquid chromatography-mass spectrometry analysis

Liquid chromatography-mass spectrometry (LC-MS) analysis was performed as previously described. Lipid extracts were analysed on an Agilent 6490 QQQ mass spectrometer with an Agilent 1290 series HPLC system. Samples from the validation cohort were analysed with other samples not part of this study as 3 batch runs, where the batch differences were adjusted by median centering with PQC samples. The median concentration of each lipid species of the PQC samples in the 3 batches was first calculated, and then used to derive a median center value for each lipid species (median center value = median of batch 1 / median of batch 1, 2 or 3). The median center value of each batch was then multiplied to the concentration of lipids in the study samples, resulting in the alignment of all batches to the first batch.

## S1.4 Data normalisation

Normalisation is a data pre-processing step that is essential for large scale analyses of multi-variable data (e.g. genomic, proteomic). This normalisation step adjusts for biases that can arise from sample preparation (e.g. sample loss, evaporation, irregular extraction efficiency, pipetting errors), biological effects (e.g. differences in water content) or biological variation (e.g. differences in individuals unrelated to disease pathology).

The lipidomic datasets from both cohorts were normalised independently according to the Probabilistic Quotient (PBQ) normalization method as previously described, and adapted from Dieterle *et al* (2006). The reference sample used in PBQ normalization was created from the mean levels of each lipid species across all the plasma samples of each cohort respectively. Final values are logarithm-2 of pmol/mL.

## S1.5 Dataset alignment and calculation of the 3-lipid signature

To determine the presence of the circulating 3LS of poor prognosis, the lipidomic data was first aligned to that of the original cohort from which the 3LS was derived using the ComBat algorithm (R package sva, v3.34.0) to remove batch differences, as the lipidomic datasets were produced by different LC-MS instruments and on a different occasion. Next, the 3LS status of each patient was calculated from a logistic regression model consisting of ceramide Cer(d18:1/24:1), sphingomyelin SM(d18:2/16:0) and phosphatidycholine PC(16:0/16:0), derived in Lin et al (2017) as follows:

$y = (3.1319 \times Cer(d18:1/24:1)) + (2.1724 \times SM(d18:2/16:0)) + (1.8593 \times PC(16:0/16:0)) – 91.217$

$p = e^{y}/(1+ e^{y})$

Patient has the poor prognostic 3LS when $p \geq 0.5$

# S2 Detailed methods for targeted cfDNA sequencing

## S2.1 Blood collection and cfDNA extraction

Peripheral blood was sampled and cell-free DNA (cfDNA) was isolated as published previously. The process of blood collection was described in S1.1. Samples were then stored at -80$^{o}$C until required for batch processing. Up to 5mL of plasma was used to extract cfDNA using the QIAamp circulating nucleic acid kit (Qiagen, Hilden, Germany), with large genomic fragments removed with AMPure XP beads (Beckman Coulter, Brea, CA, USA). DNA was quantified (Qubit 2.0 fluorometer, ThermoFisher Scientific, Waltham, Massachusetts, USA) and underwent quality assessment (Bioanalyzer 2100, Agilent Technologies, California, USA).

## S2.2 Targeted capture, sequencing and bioinformatics

### Library preparation, hybrid-capture and sequencing

Up to 40ng of extracted cfDNA was used for preparation of next-generation sequencing (NGS) libraries, a process which has been described in detail previously. Amplified DNA libraries underwent further quality assessment (Bioanalyzer 2100) and then were subsequently hybridised overnight to a targeted panel capturing exonic regions from 90-120 genes (Table S1.2.1). Captured fragments were further PCR amplified and underwent a final quality assessment (Bioanalyzer 2100), before being sequenced on the Illumina HiSeq X Ten.

**Table S2.2.1: Predicine cfDNA assay gene list**

| Predicine 90-gene cfDNA assay (Discovery cohort) | | | | | | | | | |
|---|---|---|---|---|---|---|---|---|---|
| AKT1 | ALK | APC | AR | ARAF | ARID1A | ATM | BRAF | BRCA1 | BRCA2 |
| CCND1 | CCNE1 | CD274 | CDH1 | CDK4 | CDK6 | CDKN2A | CTNNB1 | CYP2C19 | CYP2D6 |
| CYP3A4 | DDR2 | DNAJB1 | DPYD | EGFR | ERBB2 | ERBB3 | ESR1 | EZH2 | FBXW7 |
| FGFR1 | FGFR2 | FGFR3 | GNA11 | GNAQ | GNAS | GSTP1 | HRAS | IDH1 | IDH2 |
| JAK2 | JAK3 | KIT | KRAS | MAP2K1 | MAP2K2 | MAPK1 | MDM2 | MET | MLH1 |
| MPL | MSH2 | MTHFR | MTOR | MYC | MYCN | MYD88 | NF1 | NFE2L2 | NPM1 |
| NRAS | NTRK1 | NTRK3 | PDCD1LG2 | PDGFRA | PIK3CA | POLD1 | POLE | PPP2R1A | PRKACA |
| PRKD1 | PTEN | PTPN11 | RAF1 | RB1 | RET | RNF43 | ROS1 | SMAD4 | SMO |
| SPOP | STK11 | TERT | TP53 | TSC1 | TSC2 | UGT1A1 | VHL | XPC | XRCC1 |

| Predicine 120-gene cfDNA assay (Validation cohort) | | | | | | | | | |
|---|---|---|---|---|---|---|---|---|---|
| ABRAXAS1 | AKT1 | ALK | APC | AR | ARAF | ARID1A | ATM | ATR | BAP1 |
| BARD1 | BRAF | BRCA1 | BRCA2 | BRIP1 | CCND1 | CCNE1 | CD274 | CDH1 | CDK12 |
| CDK4 | CDK6 | CDKN2A | CHEK1 | CHEK2 | CTNNB1 | DDR2 | DNAJB1 | EGFR | EPCAM |
| ERBB2 | ERBB3 | ERCC1 | ERCC2 | ERCC4 | ESR1 | EZH2 | FANCA | FANCC | FANCD2 |
| FANCI | FANCL | FANCM | FBXW7 | FGFR1 | FGFR2 | FGFR3 | GNA11 | GNAQ | GNAS |
| HDAC2 | HRAS | IDH1 | IDH2 | JAK2 | JAK3 | KIT | KRAS | MAP2K1 | MAP2K2 |
| MAPK1 | MDM2 | MET | MLH1 | MPL | MRE11 | MSH2 | MSH6 | MTOR | MYC |
| MYCN | MYD88 | NBN | NF1 | NFE2L2 | NPM1 | NRAS | NTRK1 | NTRK3 | PALB2 |
| PDCD1LG2 | PDGFRA | PIK3CA | PMS2 | POLD1 | POLE | PPM1D | PPP2R1A | PPP2R2A | PRKACA |
| PRKD1 | PTEN | PTPN11 | RAD50 | RAD51 | RAD51B | RAD51C | RAD51D | RAD54L | RAF1 |
| RB1 | RECQL | RET | RNF43 | ROS1 | RPA1 | SMAD4 | SMO | SPOP | STK11 |
| TERT | TMPRSS2 | TP53 | TP53BP1 | TSC1 | TSC2 | VHL | XRCC2 | XRCC3 | XRCC4 |

### Somatic mutation identification

Consensus binary alignment map (BAM) files were derived as previously described to reduce sequencing and PCR errors. Candidate somatic variants were identified using an in-house pipeline. A variant was considered a candidate somatic mutation when all 3 criteria were met: 1) presence of at least 4 distinct fragments contained the mutation, 2) variant allelic frequency (AF) was at least 0.25%, or 0.1% for hotspot loci (as defined by COSMIC and http://www.cancerhotspots.org), and 3) variant does not appear in public databases of common germline variants (1000 genomes, ExAC, gnomAD and KAVIAR). Candidate somatic mutations were further filtered to include missense, nonsense, frameshift or splice site alterations, and to exclude benign variants and haematopoietic expansion-related variants.

### Estimation of ctDNA fraction

Circulating tumour DNA (ctDNA) was estimated based on the allele fractions of autosomal somatic mutations, using a method described previously. ctDNA fraction was dichotomized to above or below 2% for multivariable analyses, an approach that has been used in other studies involving patients with advanced prostate cancer due to challenges in accurately calculating ctDNA fraction in samples with low ctDNA abundance.

### Targeted copy number analysis

Estimation of panel-based copy number variation (CNV) has been described in greater detail previously. Briefly, in-house algorithms calculated the on-target unique fragment coverage using the consensus BAM file. The fragment was corrected for GC bias, then compared against corresponding coverage from a group of normal reference samples to estimate the significance of the copy number variant. Gains or deletions with an absolute z-score >3 and absolute CNV change above minimum gain/deletion thresholds were considered true events.

## S2.3 Analytical validation of cfDNA assay CNV detection

Details of analytical validation of sensitivity, specificity and precision of the CNV detection assay in cfDNA has been described in a previous study.

## S3 Principal components analysis (PCA) of baseline plasma lipidomic profiles in the discovery cohort

### Fig S3.1: PCA of baseline plasma lipidomic profiles in the discovery cohort, samples labelled by treatment type

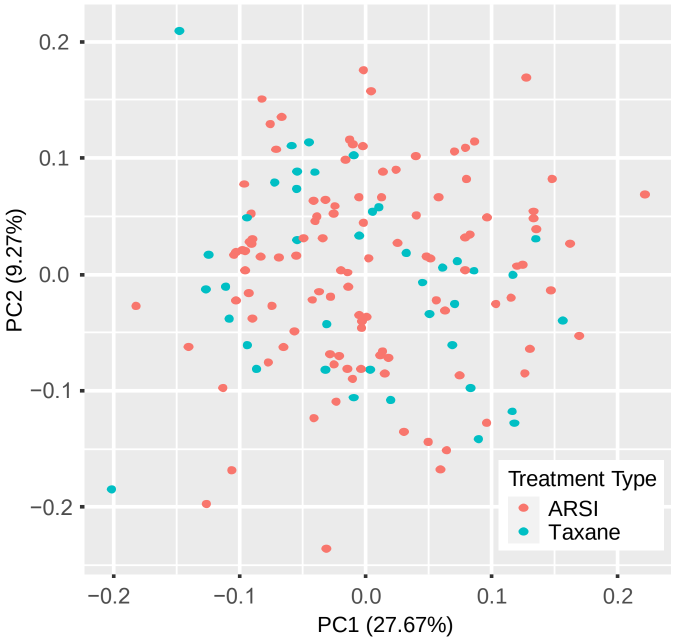


### Fig S3.2: PCA of baseline plasma lipidomic profiles in the discovery cohort, samples labelled by treatment line

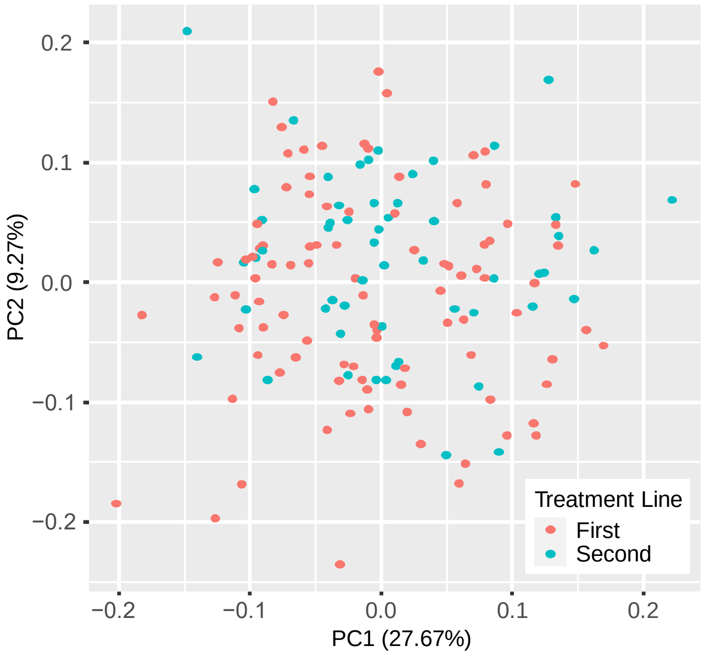

## Fig S4: CONSORT diagram

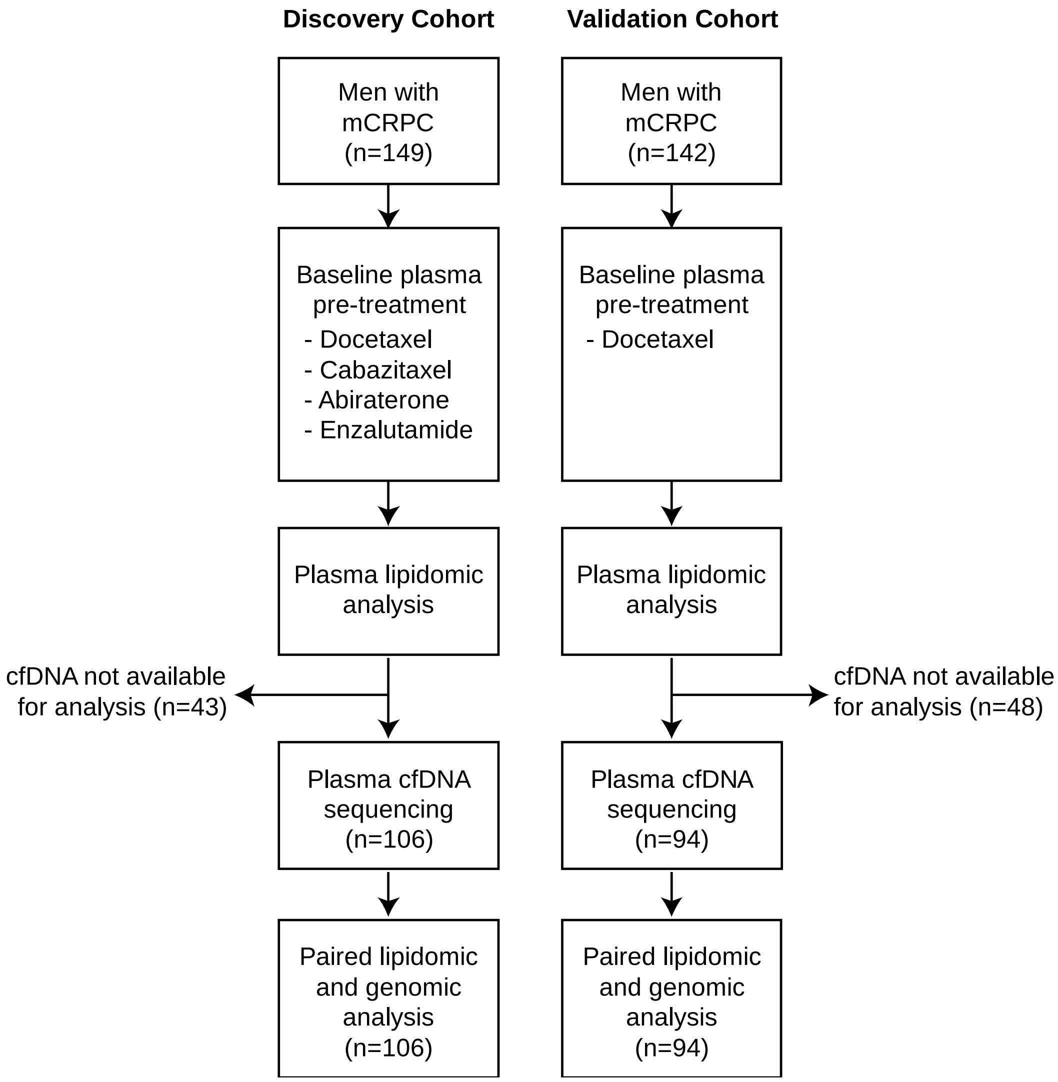

## Table S5: Patient characteristics of the discovery and validation cohorts

| | Discovery cohort With lipidomic data | Discovery cohort With lipidomic and cfDNA data | Validation cohort With lipidomic data | Validation cohort With lipidomic and cfDNA data |
|---|---|---|---|---|
| **Number of men** | 149 | 106 | 142 | 94 |
| **Median age, years [Q1,Q3]** | 73 [67,81] | 73 [66, 79] | 72 [66, 77] | 73 [66, 77] |
| **Median follow-up time, months [Q1,Q3]** | 20 [13, 28] | 18 [12,25] | 26 [14, 47] | 24 [13, 45] |
| **Prostate specific antigen at time of sample collection, ng/mL [Q1, Q3]** | 30 [11, 72] | 31 [9, 94] | 14 [3, 52] | 12 [4, 52] |
| **Deceased** | 87 [58%] | 61 [58%] | 125 [88%] | 83 [88%] |
| **Gleason grade** | | | | |
| ≤6 | 7 [5%] | 5 [5%] | 15 [11%] | 10 [11%] |
| 7 | 26 [17%] | 16 [15%] | 49 [34%] | 28 [30%] |
| 8 | 19 [13%] | 13 [12%] | 24 [17%] | 16 [17%] |
| ≥9 | 73 [49%] | 56 [53%] | 45 [32%] | 32 [34%] |
| unknown | 24 [16%] | 16 [15%] | 9 [6%] | 8 [8%] |
| **Type of metastasis** | | | | |
| Bone | 131 [88%] | 93 [88%] | 124 [87%] | 81 [86%] |
| Visceral | 15 [10%] | 13 [12%] | 10 [7%] | 7 [7%] |
| unknown | 0 [0%] | 0 [0%] | 15 [11%] | 11 [12%] |
| **Treatment at enrolment** | | | | |
| Docetaxel | 39 [26%] | 36 [34%] | 72 [51%] | 50 [53%] |
| Cabazitaxel | 1 [1%] | 0 [0%] | 0 [0%] | 0 [0%] |
| Enzalutamide | 78 [52%] | 49 [46%] | 0 [0%] | 0 [0%] |
| Abiraterone | 31 [21%] | 21 [20%] | 1 [1%] | 1 [1%] |
| Cis/carboplatin | 0 [0%] | 0 [0%] | 2 [1%] | 2 [2%] |
| Unknown | 0 [0%] | 0 [0%] | 67 [47%] | 41 [44%] |
| **Line of systemic treatment at enrolment** | | | | |
| First line | 94 [63%] | 65 [61%] | 142 [100%] | 94 [100%] |
| Second line | 55 [37%] | 41 [39%] | 0 [0%] | 0 [0%] |
| **3-Lipid Signature** | | | | |
| Yes | 54 [36%] | 43 [41%] | 56 [39%] | 39 [41%] |
| No | 95 [64%] | 63 [59%] | 86 [61%] | 55 [59%] |

Q1 = first quartile, Q3 = third quartile

## S6 Fold difference in lipid levels between men with and without genetic aberrations, as assessed with t-tests

The tables below show the fold differences of the sphingolipid levels and P-values from the t-test comparisons. The different sphingolipid isoforms have varying concentration ranges, which are displayed in Section S10.

### Table S6.1: Sphingolipids with significantly elevated levels in men with any *AR* aberration in the discovery cohort compared to men without, and their fold-change in the validation cohort.

| **Lipid** | **Discovery cohort**<br>Any *AR* aberration vs none | | **Validation cohort**<br>Any *AR* aberration vs none | |
|---|---|---|---|---|
| | **Fold** | **P-value** | **Fold** | **P-value** |
| Cer(d18:1/21:0) | 1.25 | <0.001 | 1.19 | 0.002 |
| Hex3Cer(d18:1/20:0) | 1.24 | 0.029 | 1.20 | 0.017 |
| Cer(d17:1/18:0) | 1.18 | 0.004 | 1.05 | 0.474 |
| Cer(d18:1/16:0) | 1.13 | 0.009 | 1.07 | 0.058 |
| Cer(d18:1/18:0) | 1.22 | 0.007 | 1.11 | 0.126 |
| Cer(d18:1/19:0) | 1.16 | 0.010 | 1.05 | 0.622 |
| Cer(d18:1/20:0) | 1.17 | 0.005 | 1.09 | 0.108 |
| **Cer(d18:1/24:1)** | 1.15 | 0.013 | 1.08 | 0.124 |
| Cer(d18:2/18:0) | 1.16 | 0.024 | 1.05 | 0.519 |
| Cer(d18:2/20:0) | 1.14 | 0.010 | 1.02 | 0.756 |
| Cer(d18:2/21:0) | 1.21 | 0.010 | 1.25 | 0.121 |
| Cer(d19:1/16:0) | 1.18 | 0.032 | 1.20 | 0.103 |
| Cer(d19:1/18:0) | 1.31 | 0.021 | 1.16 | 0.295 |
| Cer(d19:1/20:0) | 1.24 | 0.004 | 1.18 | 0.137 |
| HexCer(d18:1/16:0) | 1.14 | 0.028 | 1.14 | 0.057 |
| HexCer(d18:1/20:0) | 1.16 | 0.033 | 1.06 | 0.359 |
| SM(38:3) (b) | 1.06 | 0.045 | 1.17 | 0.056 |
| SM(d18:1/17:0)/SM(d17:1/18:0) | 1.13 | 0.028 | 1.03 | 0.663 |
| Sph(d16:1) | 1.10 | 0.016 | 1.04 | 0.345 |
| Sph(d18:1) | 1.14 | 0.026 | 1.01 | 0.891 |
| Cer(d20:1/26:0) | 1.11 | 0.041 | 0.93 | 0.845 |
| GM3(d18:1/20:0) | 1.19 | 0.020 | 0.97 | 0.646 |

Cer = ceramide; GM3 = GM3 ganglioside; HexCer = monohexosylceramide; Hex3Cer = trihexosylceramide; SM = sphingomyelin; Sph = sphingosine.
Cer(d18:1/24:1), which is a component of the 3-lipid signature, is bolded.

Table S6.2: Sphingolipids with significantly elevated levels in men with any *TP53* aberration in the discovery cohort compared to men without, and their fold-change in the validation cohort.

| **Lipid** | **Discovery cohort** Any *TP53* aberration vs none | | **Validation cohort** Any *TP53* aberration vs none | |
|---|---|---|---|---|
| | **Fold** | **P-value** | **Fold** | **P-value** |
| HexCer(d18:1/20:0) | 1.16 | 0.036 | 1.17 | 0.011 |
| Cer(d17:1/18:0) | 1.13 | 0.041 | 1.03 | 0.709 |
| Cer(d18:1/16:0) | 1.10 | 0.047 | 1.04 | 0.265 |
| Cer(d18:1/18:0) | 1.25 | 0.002 | 1.05 | 0.506 |
| Cer(d18:1/19:0) | 1.19 | 0.003 | 1.01 | 0.917 |
| Cer(d18:1/20:0) | 1.22 | 0.000 | 1.03 | 0.567 |
| Cer(d18:1/21:0) | 1.19 | 0.007 | 1.08 | 0.176 |
| Cer(d18:2/21:0) | 1.23 | 0.007 | 1.11 | 0.484 |
| GM3(d18:1/20:0) | 1.19 | 0.019 | 1.03 | 0.647 |
| HexCer(d18:1/18:0) | 1.16 | 0.030 | 1.13 | 0.082 |
| Hex2Cer(d18:1/20:0) | 1.21 | 0.026 | 1.15 | 0.052 |
| Cer(d16:1/18:0) | 1.15 | 0.040 | 0.90 | 0.223 |
| Cer(d16:1/20:0) | 1.13 | 0.041 | 0.90 | 0.150 |
| Cer(d17:1/20:0) | 1.15 | 0.008 | 0.96 | 0.629 |
| Cer(d18:1/22:0) | 1.11 | 0.014 | 0.99 | 0.907 |
| **Cer(d18:1/24:1)** | 1.17 | 0.004 | 0.97 | 0.555 |
| Cer(d18:2/18:0) | 1.19 | 0.009 | 0.98 | 0.838 |
| Cer(d18:2/20:0) | 1.18 | 0.001 | 0.99 | 0.825 |
| Cer(d18:2/24:1) | 1.12 | 0.042 | 0.94 | 0.253 |
| Cer(d19:1/18:0) | 1.46 | 0.001 | 0.95 | 0.709 |
| Cer(d19:1/20:0) | 1.27 | 0.002 | 0.99 | 0.920 |
| Cer(d19:1/22:0) | 1.23 | 0.018 | 0.93 | 0.407 |
| Cer(d19:1/24:1) | 1.23 | 0.029 | 0.89 | 0.138 |
| Cer(d20:1/22:0) | 1.16 | 0.011 | 0.97 | 0.717 |
| Cer(d20:1/24:1) | 1.21 | 0.013 | 0.96 | 0.648 |

Cer = ceramide; GM3 = GM3 ganglioside; HexCer = monohexosylceramide; Hex2Cer = dihexosylceramide.
Cer(d18:1/24:1), which is a component of the 3-lipid signature, is bolded.

## Table S6.3: Sphingolipids with significantly elevated levels in men with *RB1* deletion in the discovery cohort compared to men without, and their fold-change in the validation cohort.

| **Lipid** | **Discovery cohort** *RB1* deletion vs none | | **Validation cohort** *RB1* deletion vs none | |
|---|---|---|---|---|
| | **Fold** | **P-value** | **Fold** | **P-value** |
| Cer(d18:1/16:0) | 1.13 | 0.027 | 1.10 | 0.014 |
| GM3(d18:1/16:0) | 1.23 | 0.005 | 1.19 | 0.003 |
| HexCer(d18:1/16:0) | 1.16 | 0.035 | 1.21 | 0.013 |
| Cer(d17:1/18:0) | 1.20 | 0.007 | 1.00 | 0.967 |
| Cer(d18:1/14:0) | 1.17 | 0.010 | 1.09 | 0.191 |
| Cer(d18:1/18:0) | 1.27 | 0.004 | 1.07 | 0.409 |
| Cer(d18:1/20:0) | 1.24 | 0.001 | 1.08 | 0.183 |
| **Cer(d18:1/24:1)** | 1.18 | 0.007 | 1.09 | 0.102 |
| Cer(d19:1/16:0) | 1.20 | 0.040 | 1.19 | 0.168 |
| GM3(d18:1/20:0) | 1.39 | 0.000 | 1.09 | 0.235 |
| GM3(d18:1/24:1) | 1.27 | 0.014 | 1.06 | 0.365 |
| Hex2Cer(d18:1/18:0) | 1.24 | 0.035 | 1.05 | 0.502 |
| Hex2Cer(d18:1/20:0) | 1.29 | 0.010 | 1.13 | 0.125 |
| S1P(d18:1) | 1.28 | 0.002 | 1.02 | 0.728 |
| SM(38:3) (a) | 1.24 | 0.014 | 1.06 | 0.406 |
| SM(38:3) (b) | 1.09 | 0.023 | 1.07 | 0.419 |
| SM(40:3) (a) | 1.16 | 0.041 | 1.10 | 0.103 |
| SM(43:2) (b) | 1.19 | 0.004 | 1.09 | 0.061 |
| SM(44:2) | 1.20 | 0.009 | 1.05 | 0.433 |
| SM(44:3) | 1.25 | 0.007 | 1.08 | 0.144 |
| SM(d18:1/17:0)/SM(d17:1/18:0) | 1.21 | 0.003 | 1.06 | 0.411 |
| SM(d18:1/18:0)/SM(d16:1/20:0) | 1.17 | 0.031 | 1.02 | 0.669 |
| SM(d18:1/24:1) | 1.25 | 0.004 | 1.10 | 0.069 |
| SM(d19:1/24:1) | 1.23 | 0.046 | 1.04 | 0.591 |
| Sph(d16:1) | 1.16 | 0.002 | 1.04 | 0.374 |
| Sph(d18:1) | 1.34 | 0.000 | 1.11 | 0.094 |
| Sph(d18:2) | 1.20 | 0.001 | 1.05 | 0.358 |
| Cer(d20:1/24:1) | 1.31 | 0.002 | 0.94 | 0.533 |
| S1P(d18:0) | 1.32 | 0.019 | 0.97 | 0.676 |
| S1P(d18:2) | 1.24 | 0.003 | 0.98 | 0.674 |
| SM(d18:2/18:0) | 1.15 | 0.038 | 1.00 | 0.958 |

Cer = ceramide; GM3 = GM3 ganglioside; HexCer = monohexosylceramide; Hex2Cer = dihexosylceramide; SM = sphingomyelin; Sph = sphingosine; S1P = sphingosine-1-phosphate. Cer(d18:1/24:1), which is a component of the 3-lipid signature, is bolded.

## Table S6.4: Sphingolipids with significantly elevated levels in men with any PI3K pathway aberration in the discovery cohort compared to men without, and their fold-change in the validation cohort.

| Lipid | Discovery cohort<br>Any PI3K aberration vs none | | Validation cohort<br>Any PI3K aberration vs none | |
|---|---|---|---|---|
| | **Fold** | **P-value** | **Fold** | **P-value** |
| Cer(d18:1/16:0) | 1.11 | 0.023 | 1.10 | 0.012 |
| Cer(d17:1/18:0) | 1.21 | 0.001 | 1.08 | 0.333 |
| Cer(d18:1/18:0) | 1.27 | 0.001 | 1.14 | 0.082 |
| Cer(d18:1/20:0) | 1.17 | 0.004 | 1.11 | 0.060 |
| **Cer(d18:1/24:1)** | 1.14 | 0.016 | 1.07 | 0.201 |
| Cer(d18:2/18:0) | 1.19 | 0.008 | 1.13 | 0.157 |
| Cer(d19:1/18:0) | 1.33 | 0.016 | 1.23 | 0.176 |
| Cer(d19:1/20:0) | 1.19 | 0.026 | 1.15 | 0.211 |
| GM3(d18:1/20:0) | 1.25 | 0.003 | 1.08 | 0.268 |
| HexCer(d18:1/16:0) | 1.21 | 0.002 | 1.08 | 0.267 |
| HexCer(d18:1/18:0) | 1.17 | 0.024 | 1.01 | 0.836 |
| HexCer(d18:1/20:0) | 1.19 | 0.015 | 1.03 | 0.712 |
| HexCer(d18:1/24:1) | 1.17 | 0.047 | 1.09 | 0.186 |
| S1P(d18:0) | 1.35 | 0.003 | 1.01 | 0.859 |
| SM(d18:1/17:0)/SM(d17:1/18:0) | 1.15 | 0.015 | 1.09 | 0.231 |
| Sph(d18:1) | 1.13 | 0.030 | 1.02 | 0.791 |
| Sph(d18:2) | 1.13 | 0.010 | 1.04 | 0.440 |

Cer = ceramide; GM3 = GM3 ganglioside; HexCer = monohexosylceramide; SM = sphingomyelin; Sph = sphingosine; S1P = sphingosine-1-phosphate.
Cer(d18:1/24:1), which is a component of the 3-lipid signature, is bolded.

## Table S6.5: Sphingolipids with significantly elevated levels in men with any DNA repair aberration (*BRCA1/2, ATM, CHEK2*) in the discovery cohort compared to men without, and their fold-change in the validation cohort.

| Lipid | Discovery cohort<br>Any DNA repair aberration vs none | | Validation cohort<br>Any DNA repair aberration vs none | |
|---|---|---|---|---|
| | **Fold** | **P-value** | **Fold** | **P-value** |
| Cer(d17:1/18:0) | 1.14 | 0.024 | 1.05 | 0.543 |
| Cer(d18:1/18:0) | 1.17 | 0.039 | 1.07 | 0.357 |
| GM3(d18:1/20:0) | 1.19 | 0.024 | 1.02 | 0.748 |
| Hex2Cer(d18:1/20:0) | 1.28 | 0.005 | 1.10 | 0.221 |
| Sph(d18:2) | 1.12 | 0.022 | 1.08 | 0.162 |
| Cer(d20:1/22:0) | 1.18 | 0.006 | 0.81 | 0.008 |
| Cer(d20:1/23:0) | 1.12 | 0.047 | 0.85 | 0.026 |
| Cer(d20:1/24:1) | 1.20 | 0.024 | 0.92 | 0.336 |

Cer = ceramide; GM3 = GM3 ganglioside; Hex2Cer = dihexosylceramide; Sph = sphingosine.

## Table S6.6: Sphingolipids with significantly elevated levels in men with any mismatch repair (MMR) aberration (MLH1, MSH2, MSH6) in the discovery cohort compared to men without, and their fold-change in the validation cohort.

| **Lipid** | **Discovery cohort** Any MMR aberration vs none | | **Validation cohort** Any MMR aberration vs none | |
|---|---|---|---|---|
| | **Fold** | **P-value** | **Fold** | **P-value** |
| Sph(d18:1) | 1.22 | 0.025 | 1.19 | 0.147 |

Sph = sphingosine.

## Table S6.7: Sphingolipids with significantly elevated levels in men with any WNT pathway aberration (*APC, CTNNB1*) in the discovery cohort compared to men without, and their fold-change in the validation cohort.

| **Lipid** | **Discovery cohort** Any WNT pathway aberration vs none | | **Validation cohort** Any WNT pathway aberration vs none | |
|---|---|---|---|---|
| | **Fold** | **P-value** | **Fold** | **P-value** |
| Cer(d18:1/18:0) | 1.34 | 0.001 | 1.10 | 0.282 |
| Cer(d18:1/20:0) | 1.25 | 0.001 | 1.08 | 0.268 |
| **Cer(d18:1/24:1)** | 1.18 | 0.017 | 1.10 | 0.126 |
| Cer(d18:2/18:0) | 1.22 | 0.019 | 1.01 | 0.888 |
| Cer(d20:1/22:0) | 1.21 | 0.009 | 1.04 | 0.711 |
| Cer(d20:1/24:1) | 1.33 | 0.004 | 1.16 | 0.17 |
| GM3(d18:1/16:0) | 1.18 | 0.042 | 1.08 | 0.296 |
| GM3(d18:1/20:0) | 1.34 | 0.002 | 1.14 | 0.14 |
| GM3(d18:1/22:0) | 1.20 | 0.022 | 1.08 | 0.262 |
| Hex2Cer(d18:1/20:0) | 1.34 | 0.006 | 1.03 | 0.756 |
| Cer(d18:2/20:0) | 1.15 | 0.037 | 1.00 | 0.998 |

Cer = ceramide; GM3 = GM3 ganglioside; Hex2Cer = dihexosylceramide.
Cer(d18:1/24:1), which is a component of the 3-lipid signature, is bolded.

# S7 Kaplan Meier analysis of overall survival by aberration and 3-lipid signature in the discovery and validation cohorts.

The Kaplan Meier analyses below are the same as those in Figures 4-5 of the main article, but with Group 1 (3-lipid signature or aberration) displayed separately as Group 1A (3-lipid signature only) and Group 1B (aberration only).

## Fig S7.1: Kaplan Meier analysis of overall survival by *AR* aberration and 3-lipid signature in (A) the discovery cohort and (B) the validation cohort.

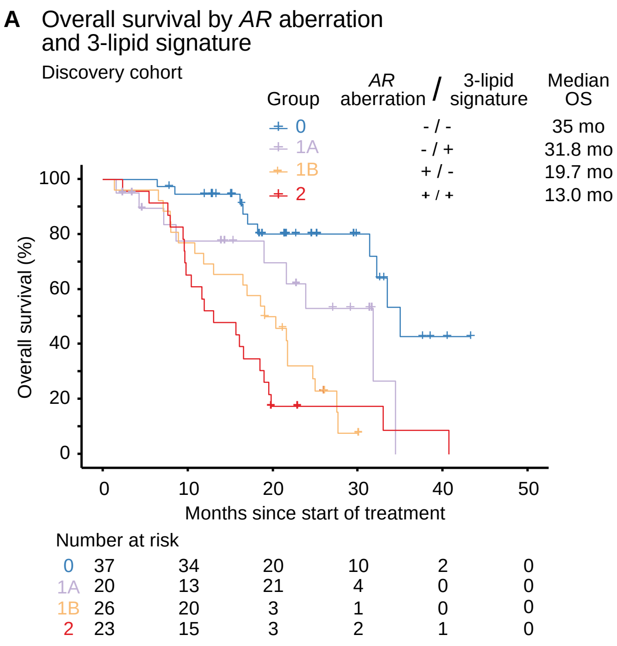


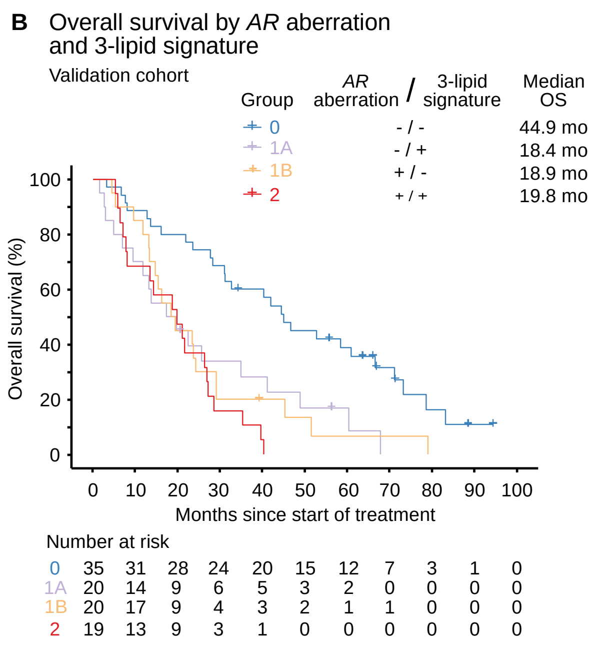


## Fig S7.2: Kaplan Meier analysis of overall survival by *TP53* aberration and 3-lipid signature in (A) the discovery cohort and (B) the validation cohort.

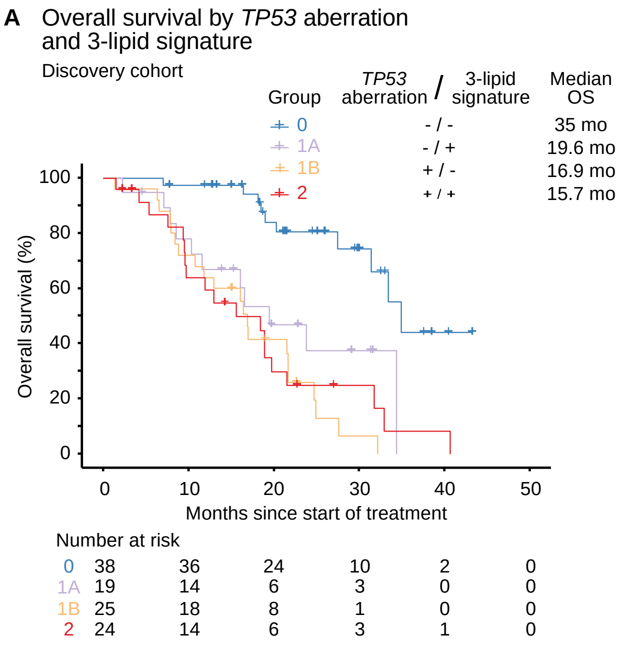


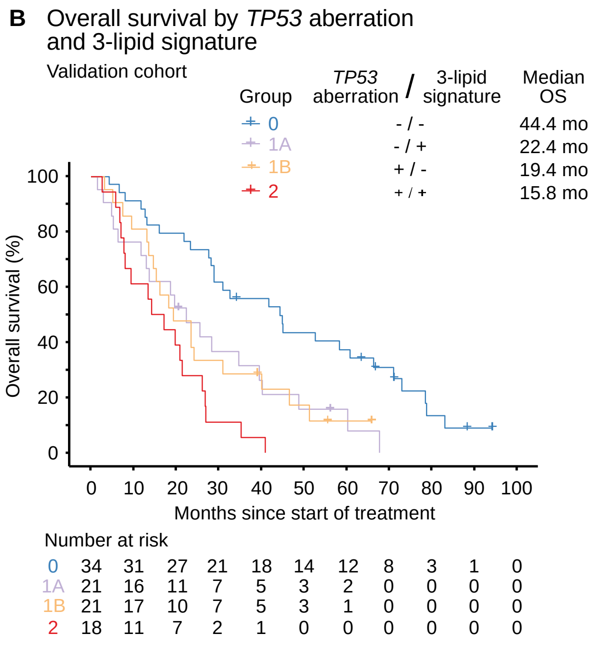

Fig S7.3: Kaplan Meier analysis of overall survival by *RB1* deletion and 3-lipid signature in (A) the discovery cohort and (B) the validation cohort.

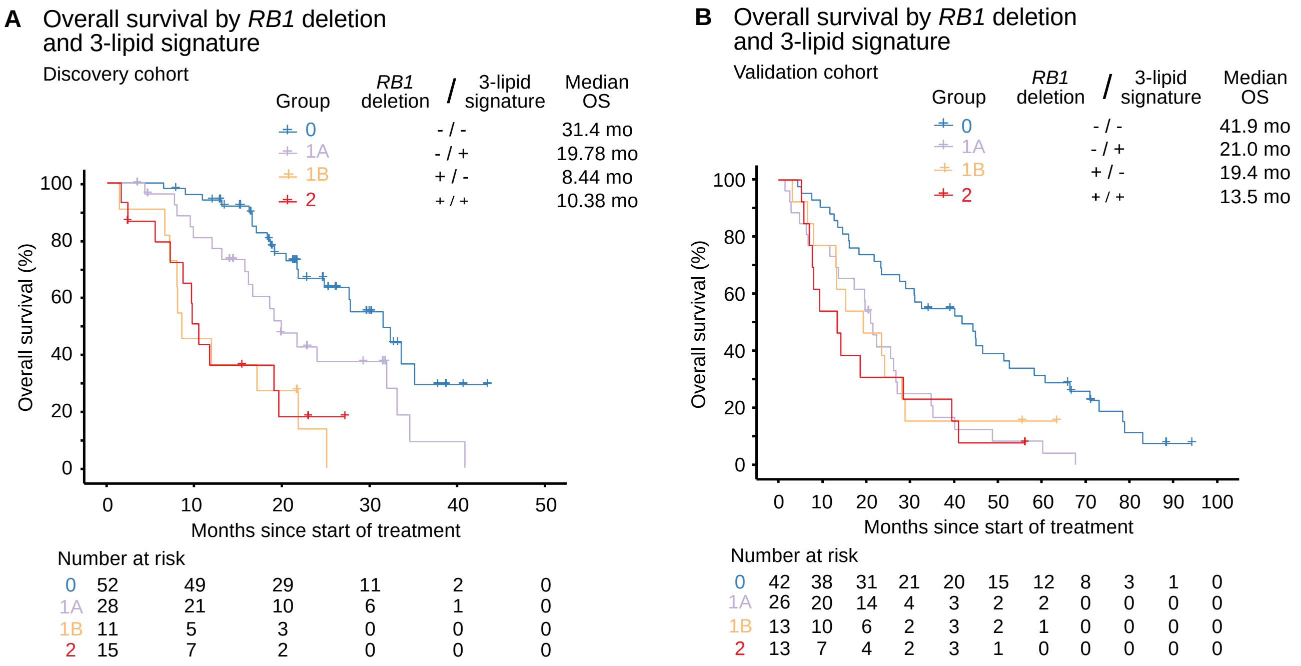


Fig S7.4: Kaplan Meier analysis of overall survival by *PI3K* aberration and 3-lipid signature in (A) the discovery cohort and (B) the validation cohort.

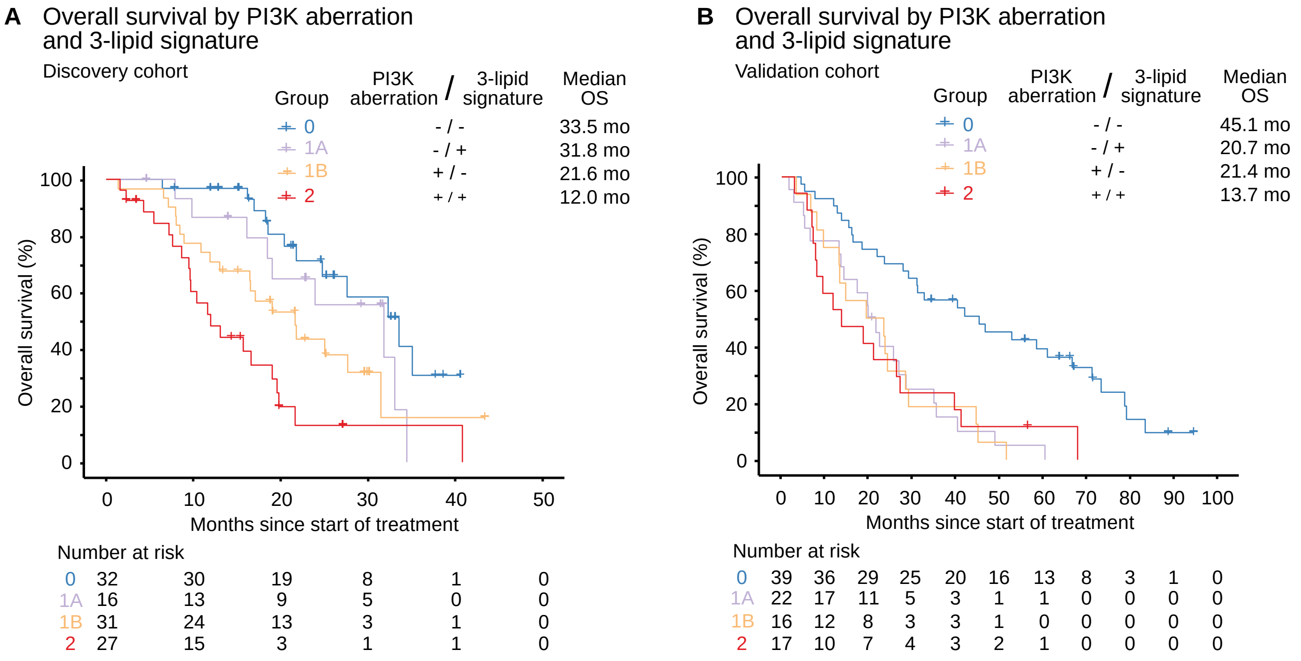

Fig S7.5: Kaplan Meier analysis of overall survival by AVPC signature and 3-lipid signature in (A) the discovery cohort and (B) the validation cohort.

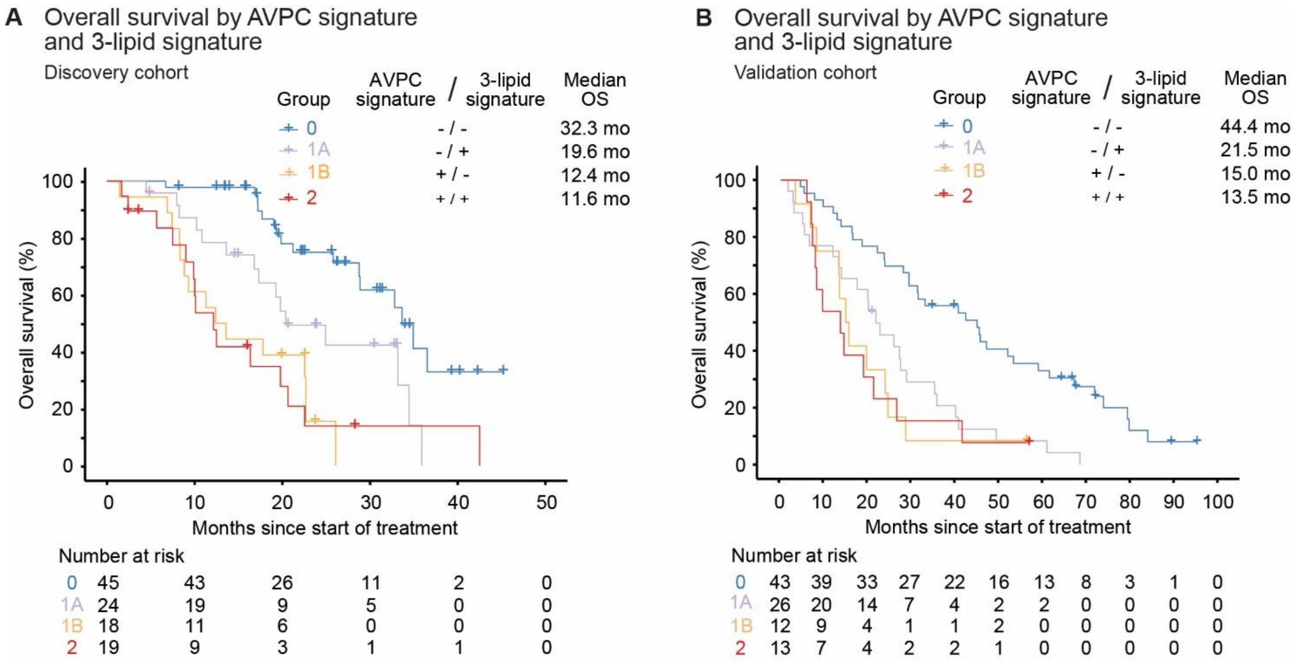

## S8 Cox proportional hazards analysis of overall survival based on the aggressive-variant prostate cancer and 3-lipid signature combination

### Table S8.1: Cox proportional hazards analysis of overall survival based on the aggressive-variant prostate cancer and 3-lipid signature combination, clinicopathologic factors and ctDNA fraction in the discovery cohort

| | Variable | Univariable Cox regression | | Multivariable Cox regression using AVPC signature and 3-lipid signature | |
|---|---|---|---|---|---|
| | | HR (95% CI) | P-value | HR (95% CI) | P-value |
| | AVPC signature and/or 3-lipid signature (Groups 1 and 2 vs 0) | 3.66 (2.05-6.54) | **<0.001** | 2.24 (1.21-4.15) | **0.01** |
| CLINCOPATHOLOGIC FACTORS | Albumin, g/L* | 0.86 (0.82-0.91) | **<0.001** | 0.89 (0.84-0.95) | **<0.001** |
| | ECOG performance status (≥2 vs 0-1) | 3.94 (1.75-8.87) | **<0.001** | 1.71 (0.72-4.06) | 0.227 |
| | Pain at baseline (present vs absent) | 1.89 (1.12-3.19) | **0.018** | 1.65 (0.94-2.89) | 0.08 |
| | Haemoglobin (<90 g/L vs ≥90 g/L) | 2.70 (0.84-8.73) | 0.096 | - | - |
| | PSA, ng/mL* | 1.00 (1.00-1.00) | 0.081 | - | - |
| | ALP, IU/L* | 1.00 (1.00-1.00) | 0.197 | - | - |
| | Treatment type (taxane vs ARSI) | 1.05 (0.62-1.79) | 0.844 | - | - |
| | Treatment line (second line vs first line) | 0.87 (0.51-1.48) | 0.597 | - | - |
| | Visceral metastases (present vs absent) | 1.59 (0.75-3.37) | 0.225 | - | - |
| | ctDNA fraction > 2% | 1.97 (1.06-3.66) | **0.032** | 2.04 (1.08-3.85) | **0.029** |

Group 0 = absence of both 3LS and the genetic aberration; Group 1 = presence of one abnormality (either 3LS or the genetic aberration); Group 2 = presence of both 3LS and the genetic aberration.
ALP = alkaline phosphatase; ARSI = androgen receptor signalling inhibitor; AVPC = aggressive-variant prostate cancer; CI = confidence interval; ctDNA = circulating tumour DNA; ECOG = Eastern Cooperative Oncology Group; HR = hazard ratio; PSA = prostate-specific antigen.
All p-values <0.05 are highlighted in bold. Only variables with p<0.05 in univariable analysis were included in multivariable analysis.
*Continuous variable

## Table S8.2: Cox proportional hazards analysis of overall survival based on the aggressive-variant prostate cancer and 3-lipid signature combination, clinicopathologic factors and ctDNA fraction in the validation cohort

| | Variable | Univariable Cox regression | | Multivariable Cox regression using AVPC signature and 3-lipid signature | |
|---|---|---|---|---|---|
| | | HR (95% CI) | P-value | HR (95% CI) | P-value |
| | AVPC signature and/or 3-lipid signature (Group 1 and 2 vs 0) | 2.94 (1.83-4.74) | **<0.001** | 2.62 (1.59-4.34) | **<0.001** |
| CLINCOPATHOLOGIC FACTORS | PSA, ng/mL* | 1.00 (1.00-1.00) | **<0.001** | 1.00 (1.00-1.00) | **<0.001** |
| | ALP, IU/L* | 1.00 (1.00-1.00) | **0.002** | 1.00 (1.00-1.00) | 0.074 |
| | ctDNA fraction > 2% | 1.91 (1.18-3.08) | **0.008** | 1.47 (0.88-2.44) | 0.144 |

Group 0 = absence of both 3LS and the genetic aberration; Group 1 = presence of one abnormality (either 3LS or the genetic aberration); Group 2 = presence of both 3LS and the genetic aberration.
ALP = alkaline phosphatase; AVPC = aggressive-variant prostate cancer; CI = confidence interval; ctDNA = circulating tumour DNA; HR = hazard ratio; PSA = prostate-specific antigen.
All p-values <0.05 are highlighted in bold. Only variables with p<0.05 in univariable analysis were included in multivariable analysis.
*Continuous variable

# S9 Bivariable Cox proportional hazard analyses

## Table S9.1: Bivariable Cox proportional hazards analysis of overall survival based on 3-lipid signature and *AR* aberration

| **Variable** | **Univariable Cox regression** | | **Bivariable Cox regression** | |
|---|---|---|---|---|
| | **HR (95% CI)** | **P-value** | **HR (95% CI)** | **P-value** |
| DISCOVERY COHORT | | | | |
| 3-lipid signature | 1.95 (1.18-3.22) | 0.01 | 1.58 (0.94-2.64) | 0.085 |
| *AR* aberration | 3.87 (2.21-6.77) | <0.001 | 3.54 (2.01-6.21) | <0.001 |
| VALIDATION COHORT | | | | |
| 3-lipid signature | 2.23 (1.41-3.52) | <0.001 | 2.00 (1.26-3.19) | 0.003 |
| *AR* aberration | 2.26 (1.43-3.58) | <0.001 | 2.04 (1.28-3.26) | 0.003 |

## Table S9.2: Bivariable Cox proportional hazards analysis of overall survival based on 3-lipid signature and *TP53* aberration

| **Variable** | **Univariable Cox regression** | | **Bivariable Cox regression** | |
|---|---|---|---|---|
| | **HR (95% CI)** | **P-value** | **HR (95% CI)** | **P-value** |
| DISCOVERY COHORT | | | | |
| 3-lipid signature | 1.95 (1.18-3.22) | 0.01 | 1.40 (0.83-2.37) | 0.209 |
| *TP53* aberration | 3.55 (2.07-6.08) | <0.001 | 3.21 (1.84-5.62) | <0.001 |
| VALIDATION COHORT | | | | |
| 3-lipid signature | 2.23 (1.41-3.52) | <0.001 | 2.17 (1.37-3.44) | <0.001 |
| *TP53* aberration | 2.18 (1.37-3.47) | 0.001 | 2.12 (1.32-3.39) | 0.002 |

## Table S9.3: Bivariable Cox proportional hazards analysis of overall survival based on 3-lipid signature and *RB1* deletion

| **Variable** | **Univariable Cox regression** | | **Bivariable Cox regression** | |
|---|---|---|---|---|
| | **HR (95% CI)** | **P-value** | **HR (95% CI)** | **P-value** |
| DISCOVERY COHORT | | | | |
| 3-lipid signature | 1.95 (1.18-3.22) | 0.01 | 1.58 (0.94-2.66) | 0.083 |
| *RB1* deletion | 4.10 (2.33-7.22) | <0.001 | 3.66 (2.05-6.54) | <0.001 |
| VALIDATION COHORT | | | | |
| 3-lipid signature | 2.23 (1.41-3.52) | <0.001 | 2.09 (1.32-3.32) | 0.002 |
| *RB1* deletion | 1.79 (1.09-2.94) | 0.021 | 1.57 (0.95-2.59) | 0.079 |

## Table S9.4: Bivariable Cox proportional hazards analysis of overall survival based on 3-lipid signature and PI3K aberration

| Variable | Univariable Cox regression | | Bivariable Cox regression | |
|---|---|---|---|---|
| | HR (95% CI) | P-value | HR (95% CI) | P-value |
| DISCOVERY COHORT | | | | |
| 3-lipid signature | 1.95 (1.18-3.22) | 0.01 | 1.89 (1.14-3.14) | 0.014 |
| PI3K aberration | 2.66 (1.55-4.58) | <0.001 | 2.61 (1.52-4.49) | <0.001 |
| VALIDATION COHORT | | | | |
| 3-lipid signature | 2.23 (1.41-3.52) | <0.001 | 1.91 (1.19-3.07) | 0.007 |
| PI3K aberration | 2.11 (1.33-3.34) | 0.002 | 1.75 (1.08-2.82) | 0.022 |

PI3K = phosphatidylinositol-3-kinase.

# S10 Plasma concentrations of sphingolipids in the discovery and validation cohorts.

Sphingolipids are lipids with a sphingoid base of which the d18:1 isoform is the major isoform present in plasma.

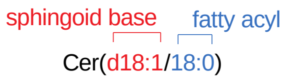


The most abundant ceramide species measured in the plasma of our cohorts were Cer(d18:1/24:0), Cer(d18:1/24:1), Cer(d18:1/22:0) and Cer(d18:1/23:0) (Figure S10.1), of which Cer(d18:1/24:1) is the ceramide in the 3LS. This ceramide is one of the ceramides with significantly elevated levels among patients with genetic aberrations in AR, TP53 or RBI compared to those without the respective aberration. The plasma concentration ranges (pre-treatment) of each individual sphingolipid species are displayed in Table S10.1.

## Fig S10.1: Abundance of ceramides in the plasma (pre-treatment) of the Discovery and Validation cohorts.

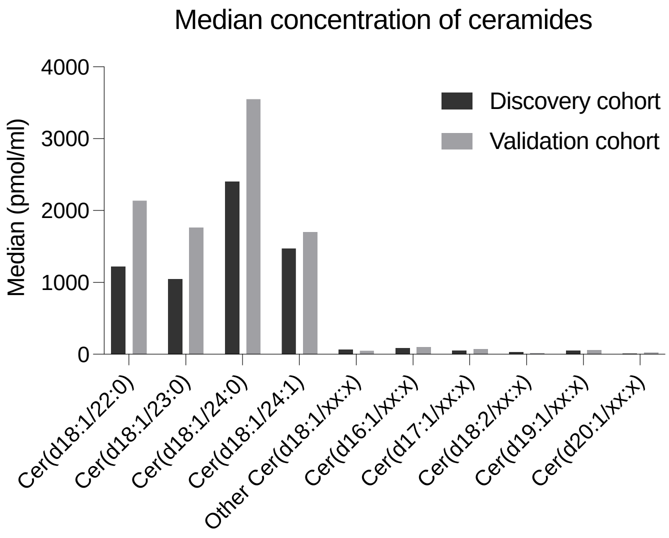

## Table S10.1: Concentration range of sphingolipids in plasma (baseline) of the Discovery and Validation cohorts.

| | Sphingolipid in plasma | Discovery cohort (pmol/ml) | | | Validation cohort (pmol/ml) | | |
|---|---|---|---|---|---|---|---|
| | | Median | Min | Max | Median | Min | Max |
| 1 | Cer(d16:1/16:0) | 33.5 | 13.2 | 77.3 | 25.6 | 4.2 | 85.6 |
| 2 | Cer(d16:1/18:0) | 46.8 | 14.7 | 89.0 | 30.8 | 6.1 | 117.7 |
| 3 | Cer(d16:1/20:0) | 47.8 | 19.6 | 98.5 | 29.4 | 7.9 | 149.7 |
| 4 | Cer(d16:1/22:0) | 96.1 | 32.9 | 232.8 | 161.4 | 43.9 | 1424.3 |
| 5 | Cer(d16:1/23:0) | 84.0 | 21.9 | 232.8 | 133.1 | 39.6 | 830.8 |
| 6 | Cer(d16:1/24:0) | 286.2 | 89.4 | 753.8 | 391.9 | 60.0 | 2938.3 |
| 7 | Cer(d16:1/24:1) | 82.4 | 25.0 | 179.4 | 101.5 | 33.3 | 550.1 |
| 8 | Cer(d17:1/16:0) | 16.7 | 6.0 | 32.5 | 17.4 | 6.8 | 70.2 |
| 9 | Cer(d17:1/18:0) | 10.9 | 4.4 | 21.5 | 12.5 | 6.3 | 30.1 |
| 10 | Cer(d17:1/20:0) | 9.2 | 3.7 | 18.4 | 5.2 | 2.1 | 18.4 |
| 11 | Cer(d17:1/22:0) | 52.7 | 14.5 | 111.9 | 85.1 | 25.8 | 473.3 |
| 12 | Cer(d17:1/23:0) | 60.0 | 15.6 | 156.1 | 70.0 | 21.7 | 405.6 |
| 13 | Cer(d17:1/24:0) | 167.3 | 45.6 | 376.4 | 215.6 | 49.0 | 855.8 |
| 14 | Cer(d17:1/24:1) | 96.7 | 25.7 | 177.6 | 109.3 | 47.8 | 444.4 |
| 15 | Cer(d18:0/16:0) | N/A | N/A | N/A | 29.9 | 13.8 | 71.4 |
| 16 | Cer(d18:0/18:0) | N/A | N/A | N/A | 9.7 | 3.0 | 29.5 |
| 17 | Cer(d18:0/20:0) | N/A | N/A | N/A | 46.7 | 21.2 | 201.3 |
| 18 | Cer(d18:0/22:0) | N/A | N/A | N/A | 252.7 | 70.4 | 1224.8 |
| 19 | Cer(d18:0/24:0) | N/A | N/A | N/A | 179.9 | 46.3 | 890.5 |
| 20 | Cer(d18:0/24:1) | N/A | N/A | N/A | 141.7 | 42.7 | 1014.1 |
| 21 | Cer(d18:1/14:0) | 9.3 | 4.2 | 22.0 | 8.3 | 2.4 | 23.5 |
| 22 | Cer(d18:1/16:0) | 306.8 | 176.6 | 506.3 | 257.5 | 129.0 | 821.3 |
| 23 | Cer(d18:1/17:0) | N/A | N/A | N/A | 14.4 | 7.2 | 29.2 |
| 24 | Cer(d18:1/18:0) | 110.1 | 42.9 | 325.8 | 88.4 | 32.7 | 304.2 |
| 25 | Cer(d18:1/19:0) | 7.2 | 3.8 | 14.3 | 3.3 | 0.7 | 11.0 |
| 26 | Cer(d18:1/20:0) | 123.1 | 66.6 | 254.7 | 169.6 | 72.0 | 780.1 |
| 27 | Cer(d18:1/21:0) | 28.5 | 11.7 | 57.9 | 48.9 | 23.1 | 223.1 |
| 28 | Cer(d18:1/22:0) | 1220.0 | 585.0 | 2178.5 | 2136.8 | 668.8 | 13258.6 |
| 29 | Cer(d18:1/23:0) | 1049.8 | 409.8 | 2103.9 | 1763.2 | 584.6 | 7590.7 |
| 30 | Cer(d18:1/24:0) | 2405.1 | 971.5 | 4160.9 | 3553.1 | 799.5 | 16744.0 |
| 31 | Cer(d18:1/24:1) | 1471.4 | 570.9 | 2994.7 | 1700.5 | 789.4 | 5881.2 |
| 32 | Cer(d18:1/26:0) | 49.4 | 22.1 | 87.4 | 46.0 | 12.1 | 106.2 |
| 33 | Cer(d18:2/14:0) | 2.4 | 0.4 | 6.0 | 2.6 | 0.5 | 8.1 |
| 34 | Cer(d18:2/16:0) | 36.6 | 16.0 | 72.2 | 37.0 | 13.9 | 94.5 |
| 35 | Cer(d18:2/17:0) | 1.3 | 0.1 | 3.5 | 0.5 | 0.0 | 1.7 |
| 36 | Cer(d18:2/18:0) | 32.4 | 12.0 | 76.1 | 18.6 | 4.9 | 72.9 |
| 37 | Cer(d18:2/20:0) | 24.4 | 12.8 | 45.6 | 18.4 | 6.7 | 89.1 |
| 38 | Cer(d18:2/21:0) | 4.0 | 1.2 | 11.3 | 3.3 | 1.4 | 15.9 |
| 39 | Cer(d18:2/22:0) | 179.1 | 76.3 | 366.8 | 339.2 | 88.9 | 2423.7 |
| 40 | Cer(d18:2/23:0) | 194.6 | 53.7 | 479.0 | 326.0 | 93.2 | 1789.1 |
| 41 | Cer(d18:2/24:0) | 555.2 | 222.2 | 1274.2 | 889.7 | 139.7 | 4484.8 |
| 42 | Cer(d18:2/24:1) | 232.0 | 84.8 | 567.0 | 270.1 | 131.5 | 1175.6 |

| | | | | | | | |
|---|---|---|---|---|---|---|---|
| 43 | Cer(d18:2/26:0) | 9.1 | 3.1 | 22.9 | 8.9 | 1.3 | 25.2 |
| 44 | Cer(d19:1/16:0) | 1.3 | 0.3 | 2.9 | 2.6 | 0.3 | 7.0 |
| 45 | Cer(d19:1/18:0) | 6.4 | 1.0 | 23.8 | 5.1 | 0.6 | 23.4 |
| 46 | Cer(d19:1/20:0) | 8.7 | 2.5 | 24.8 | 9.7 | 3.2 | 31.4 |
| 47 | Cer(d19:1/22:0) | 94.5 | 12.6 | 248.0 | 109.5 | 23.1 | 422.9 |
| 48 | Cer(d19:1/23:0) | 92.7 | 13.6 | 312.4 | 113.9 | 25.8 | 370.6 |
| 49 | Cer(d19:1/24:0) | 217.9 | 39.0 | 525.4 | 268.6 | 33.6 | 1301.3 |
| 50 | Cer(d19:1/24:1) | 192.3 | 21.1 | 540.8 | 156.7 | 53.6 | 463.9 |
| 51 | Cer(d19:1/26:0) | 7.4 | 4.2 | 14.2 | 4.5 | 1.8 | 11.0 |
| 52 | Cer(d20:1/22:0) | 12.9 | 5.9 | 24.7 | 27.2 | 6.0 | 84.9 |
| 53 | Cer(d20:1/23:0) | 8.1 | 3.2 | 16.6 | 16.3 | 1.9 | 39.8 |
| 54 | Cer(d20:1/24:0) | 20.7 | 9.8 | 40.6 | 33.6 | 5.2 | 103.7 |
| 55 | Cer(d20:1/24:1) | 15.6 | 6.9 | 51.9 | 24.0 | 5.2 | 70.9 |
| 56 | Cer(d20:1/26:0) | 6.1 | 3.5 | 11.0 | 4.2 | 2.0 | 8.6 |
| 57 | Cer(m18:0/20:0) | 8.1 | 2.3 | 32.3 | 27.4 | 10.0 | 179.8 |
| 58 | Cer(m18:0/22:0) | 22.1 | 6.9 | 77.8 | 74.3 | 23.6 | 455.9 |
| 59 | Cer(m18:0/23:0) | 11.1 | 2.0 | 44.7 | 34.1 | 8.7 | 186.3 |
| 60 | Cer(m18:0/24:0) | 27.3 | 4.9 | 95.8 | 77.1 | 20.0 | 379.9 |
| 61 | Cer(m18:0/24:1) | 21.1 | 4.9 | 74.1 | 54.1 | 14.0 | 214.1 |
| 62 | Cer(m18:1/18:0) | 4.5 | 1.1 | 14.5 | 12.4 | 2.4 | 44.6 |
| 63 | Cer(m18:1/20:0) | 24.5 | 8.5 | 86.9 | 93.8 | 23.4 | 437.2 |
| 64 | Cer(m18:1/22:0) | 48.3 | 12.3 | 163.2 | 193.1 | 39.4 | 1023.0 |
| 65 | Cer(m18:1/23:0) | 20.8 | 4.7 | 85.6 | 80.0 | 18.1 | 336.0 |
| 66 | Cer(m18:1/24:0) | 67.8 | 19.2 | 247.5 | 209.9 | 38.8 | 894.9 |
| 67 | Cer(m18:1/24:1) | 38.9 | 8.7 | 203.2 | 133.3 | 25.3 | 615.4 |
| 68 | Cer1P(d18:1/16:0) | 2.9 | 1.1 | 7.0 | 5.4 | 2.3 | 10.8 |
| 69 | dhCer(d18:0/16:0) | 7.8 | 4.3 | 13.1 | N/A | N/A | N/A |
| 70 | dhCer(d18:0/18:0) | 2.6 | 1.0 | 5.6 | N/A | N/A | N/A |
| 71 | dhCer(d18:0/20:0) | 9.0 | 4.9 | 18.9 | N/A | N/A | N/A |
| 72 | dhCer(d18:0/22:0) | 32.0 | 14.9 | 75.7 | N/A | N/A | N/A |
| 73 | dhCer(d18:0/24:0) | 28.2 | 9.6 | 79.6 | N/A | N/A | N/A |
| 74 | dhCer(d18:0/24:1) | 24.1 | 9.7 | 76.4 | N/A | N/A | N/A |
| 75 | GM1(d18:1/16:0) | 44.1 | 21.9 | 154.5 | 46.7 | 23.2 | 91.5 |
| 76 | GM3(d18:1/16:0) | 343.4 | 148.6 | 703.3 | 312.1 | 171.3 | 1051.4 |
| 77 | GM3(d18:1/18:0) | 116.0 | 38.6 | 218.9 | 136.2 | 46.4 | 767.4 |
| 78 | GM3(d18:1/20:0) | 363.6 | 142.0 | 1035.4 | 136.7 | 58.8 | 315.3 |
| 79 | GM3(d18:1/22:0) | 355.9 | 141.4 | 964.5 | 387.1 | 143.1 | 829.4 |
| 80 | GM3(d18:1/24:0) | 119.6 | 37.6 | 290.7 | 118.5 | 42.8 | 314.7 |
| 81 | GM3(d18:1/24:1) | 277.7 | 45.0 | 801.2 | 262.1 | 108.1 | 677.4 |
| 82 | HexCer(d16:1/18:0) | 34.4 | 4.5 | 112.2 | 17.6 | 5.5 | 51.4 |
| 83 | HexCer(d16:1/20:0) | 40.2 | 9.8 | 94.3 | 23.5 | 8.6 | 62.0 |
| 84 | HexCer(d16:1/22:0) | 56.2 | 17.2 | 173.8 | 47.6 | 19.6 | 197.4 |
| 85 | HexCer(d16:1/24:0) | 78.5 | 21.7 | 279.5 | 46.7 | 17.9 | 193.6 |
| 86 | HexCer(d18:1/16:0) | 826.3 | 350.4 | 1614.7 | 506.6 | 246.7 | 1287.4 |
| 87 | HexCer(d18:1/18:0) | 112.0 | 40.6 | 254.2 | 77.2 | 29.1 | 341.6 |
| 88 | HexCer(d18:1/20:0) | 106.4 | 42.6 | 230.7 | 73.6 | 28.3 | 243.8 |

| | | | | | | | |
|---|---|---|---|---|---|---|---|
| 89 | HexCer(d18:1/22:0) | 583.4 | 281.9 | 1225.6 | 496.4 | 138.0 | 1844.2 |
| 90 | HexCer(d18:1/24:0) | 1362.7 | 560.1 | 2491.1 | 1070.3 | 296.1 | 2951.8 |
| 91 | HexCer(d18:1/24:1) | 667.7 | 274.4 | 1783.6 | 450.4 | 150.4 | 1270.0 |
| 92 | HexCer(d18:2/18:0) | 15.5 | 1.2 | 41.3 | 13.8 | 5.1 | 46.5 |
| 93 | HexCer(d18:2/20:0) | 18.3 | 6.4 | 46.0 | 11.5 | 3.7 | 44.4 |
| 94 | HexCer(d18:2/22:0) | 73.7 | 27.6 | 150.1 | 59.4 | 22.4 | 295.0 |
| 95 | HexCer(d18:2/24:0) | 225.6 | 94.6 | 531.4 | 168.9 | 58.1 | 644.9 |
| 96 | Hex2Cer(d16:1/16:0) | 49.1 | 11.4 | 129.0 | 109.2 | 36.2 | 288.8 |
| 97 | Hex2Cer(d16:1/24:1) | 31.4 | 10.9 | 94.5 | 22.1 | 4.5 | 55.3 |
| 98 | Hex2Cer(d18:1/16:0) | 1213.0 | 297.6 | 3630.4 | 1297.4 | 492.9 | 3189.2 |
| 99 | Hex2Cer(d18:1/18:0) | 17.7 | 3.4 | 37.2 | 18.3 | 5.2 | 57.6 |
| 100 | Hex2Cer(d18:1/20:0) | 26.6 | 4.6 | 80.9 | 25.4 | 9.3 | 70.8 |
| 101 | Hex2Cer(d18:1/22:0) | 131.7 | 52.4 | 296.5 | 116.6 | 49.1 | 330.8 |
| 102 | Hex2Cer(d18:1/24:0) | 258.8 | 107.3 | 589.8 | 205.9 | 89.7 | 604.4 |
| 103 | Hex2Cer(d18:1/24:1) | 365.9 | 99.2 | 858.9 | 262.9 | 112.2 | 715.0 |
| 104 | Hex2Cer(d18:2/16:0) | 61.2 | 22.5 | 135.6 | 77.0 | 28.7 | 212.8 |
| 105 | Hex2Cer(d18:2/24:1) | 103.9 | 21.8 | 241.4 | 68.5 | 24.3 | 189.2 |
| 106 | Hex3Cer(d18:1/16:0) | 509.0 | 253.8 | 1097.6 | 770.2 | 432.8 | 1646.8 |
| 107 | Hex3Cer(d18:1/18:0) | 137.5 | 50.7 | 436.8 | 134.2 | 38.0 | 335.8 |
| 108 | Hex3Cer(d18:1/20:0) | 29.8 | 6.6 | 83.7 | 35.2 | 12.3 | 118.8 |
| 109 | Hex3Cer(d18:1/22:0) | 107.4 | 34.4 | 274.3 | 132.9 | 48.6 | 377.3 |
| 110 | Hex3Cer(d18:1/24:0) | 51.4 | 21.1 | 114.9 | 72.3 | 27.5 | 187.8 |
| 111 | Hex3Cer(d18:1/24:1) | 201.4 | 69.5 | 485.1 | 191.8 | 95.5 | 616.6 |
| 112 | SM(34:3) | 48.1 | 22.0 | 86.8 | 93.9 | 41.8 | 220.4 |
| 113 | SM(35:2) (b) | 28.8 | 11.0 | 63.5 | 140.8 | 59.3 | 345.0 |
| 114 | SM(37:1) | 885.2 | 240.0 | 1695.5 | 1025.9 | 454.3 | 1796.1 |
| 115 | SM(37:2) | 209.7 | 77.9 | 403.1 | 254.6 | 126.7 | 601.6 |
| 116 | SM(38:3) (a) | 233.4 | 78.3 | 566.0 | 376.4 | 145.1 | 684.7 |
| 117 | SM(38:3) (b) | 569.4 | 426.9 | 743.0 | 168.8 | 84.2 | 315.0 |
| 118 | SM(40:3) (a) | 603.4 | 257.7 | 1190.6 | 1297.4 | 549.8 | 3578.5 |
| 119 | SM(40:3) (b) | 649.5 | 298.2 | 1483.0 | 1011.6 | 528.8 | 1996.8 |
| 120 | SM(41:0) | 143.3 | 62.7 | 441.3 | 300.9 | 98.6 | 1490.9 |
| 121 | SM(41:1) (a) | 791.6 | 139.1 | 1672.9 | 577.7 | 122.9 | 1730.6 |
| 122 | SM(43:1) | 863.9 | 255.4 | 2108.6 | 1225.3 | 285.6 | 2971.5 |
| 123 | SM(43:2) (b) | 648.1 | 364.0 | 1206.3 | 492.4 | 237.7 | 1127.7 |
| 124 | SM(43:2) (c) | 192.7 | 77.2 | 325.8 | 353.4 | 119.4 | 646.4 |
| 125 | SM(44:1) | 168.3 | 75.0 | 298.3 | 188.0 | 59.6 | 388.3 |
| 126 | SM(44:2) | 270.7 | 129.7 | 513.7 | 353.6 | 152.5 | 801.4 |
| 127 | SM(44:3) | 266.4 | 110.9 | 569.9 | 339.4 | 169.8 | 687.2 |
| 128 | SM(d16:1/19:0) | 1093.5 | 227.1 | 2524.3 | 1038.6 | 413.1 | 2276.8 |
| 129 | SM(d16:1/23:0)/ SM(d17:1/22:0) | 2446.2 | 752.2 | 5665.6 | 4621.5 | 1761.7 | 13035.5 |
| 130 | SM(d16:1/24:1) | 3715.1 | 1866.7 | 7665.0 | 4939.9 | 1688.4 | 11696.3 |
| 131 | SM(d17:1/14:0) | 169.6 | 48.9 | 449.9 | 210.2 | 101.8 | 642.9 |
| 132 | SM(d17:1/16:0) | 8260.4 | 3605.9 | 16028.9 | 6520.6 | 2605.5 | 19327.1 |
| 133 | SM(d17:1/24:1) | 2437.6 | 1162.3 | 4527.2 | 3297.7 | 1417.7 | 6543.5 |

| | | | | | | | |
|---|---|---|---|---|---|---|---|
| 134 | SM(d18:0/14:0) | 127.9 | 33.9 | 489.1 | 204.2 | 114.7 | 811.0 |
| 135 | SM(d18:0/16:0) | 5728.4 | 2787.4 | 16980.8 | 5554.8 | 3059.7 | 25731.7 |
| 136 | SM(d18:0/22:0) | 304.6 | 96.2 | 1120.0 | 499.6 | 104.2 | 2868.6 |
| 137 | SM(d18:1/14:0)/ SM(d16:1/16:0) | 7353.8 | 3253.9 | 16495.6 | 8952.8 | 4635.8 | 29381.9 |
| 138 | SM(d18:1/16:0) | 79303.3 | 32729.1 | 134579.2 | 103120.6 | 55360.8 | 184453.8 |
| 139 | SM(d18:1/17:0)/ SM(d17:1/18:0) | 2631.2 | 1103.9 | 4910.3 | 1503.0 | 736.1 | 3256.4 |
| 140 | SM(d18:1/18:0)/ SM(d16:1/20:0) | 13663.1 | 6271.0 | 37707.4 | 18420.5 | 8288.9 | 56466.7 |
| 141 | SM(d18:1/20:0)/ SM(d16:1/22:0) | 6044.3 | 2832.9 | 14779.0 | 11378.9 | 3906.9 | 37222.8 |
| 142 | SM(d18:1/22:0)/ SM(d16:1/24:0) | 18981.8 | 7468.2 | 35819.2 | 29834.7 | 9366.4 | 90811.9 |
| 143 | SM(d18:1/23:0)/ SM(d17:1/24:0) | 8159.4 | 2901.0 | 16405.3 | 12398.5 | 3326.1 | 33077.0 |
| 144 | SM(d18:1/24:0) | 11397.2 | 5527.6 | 23784.7 | 21639.5 | 6445.1 | 59810.7 |
| 145 | SM(d18:1/24:1) | 28616.3 | 12923.4 | 64957.5 | 32960.6 | 16551.9 | 86692.5 |
| 146 | SM(d18:2/14:0) | 594.3 | 229.0 | 1323.6 | 830.6 | 440.0 | 2386.9 |
| 147 | SM(d18:2/16:0) | 8198.6 | 4018.1 | 15488.3 | 14162.4 | 6320.4 | 43119.8 |
| 148 | SM(d18:2/17:0) | 271.8 | 112.5 | 516.0 | 383.2 | 140.0 | 1014.9 |
| 149 | SM(d18:2/18:0) | 7925.9 | 3807.9 | 18444.8 | 11693.9 | 5188.3 | 31823.4 |
| 150 | SM(d18:2/18:1) | 312.0 | 119.2 | 638.0 | 786.9 | 331.5 | 1994.0 |
| 151 | SM(d18:2/20:0) | 4278.0 | 1934.3 | 6886.8 | 4623.7 | 2059.3 | 10472.9 |
| 152 | SM(d18:2/22:0) | 6826.4 | 2698.2 | 13256.2 | 13650.9 | 5463.4 | 39608.1 |
| 153 | SM(d18:2/23:0) | 4190.1 | 1353.9 | 7483.4 | 6249.4 | 2685.3 | 16916.2 |
| 154 | SM(d18:2/24:0) | 7349.6 | 3483.4 | 15062.8 | 11618.3 | 4263.0 | 26243.3 |
| 155 | SM(d19:1/24:1) | 1637.7 | 320.1 | 3961.3 | 1405.6 | 384.7 | 4436.4 |
| 156 | Sph(d16:1) | 45.2 | 33.4 | 62.6 | 18.1 | 13.0 | 25.1 |
| 157 | Sph(d18:1) | 46.4 | 25.6 | 143.1 | 61.0 | 40.4 | 106.3 |
| 158 | Sph(d18:2) | 18.5 | 11.8 | 28.4 | 17.1 | 11.3 | 33.6 |
| 159 | S1P(d16:1) | 156.8 | 65.4 | 309.2 | 115.7 | 66.0 | 204.4 |
| 160 | S1P(d17:1) | 42.9 | 10.1 | 94.1 | N/A | N/A | N/A |
| 161 | S1P(d18:0) | 46.5 | 11.7 | 169.2 | 71.7 | 39.2 | 149.2 |
| 162 | S1P(d18:1) | 773.3 | 386.7 | 1822.3 | 484.4 | 303.7 | 885.6 |
| 163 | S1P(d18:2) | 246.7 | 118.3 | 570.2 | 145.2 | 96.4 | 258.9 |

N/A, not measured
